# Central body fatness is a stronger predictor of cancer risk than overall body size

**DOI:** 10.1038/s41467-018-08159-w

**Published:** 2019-01-22

**Authors:** Amanda M. Barberio, Asalah Alareeki, Benjamin Viner, Joy Pader, Jennifer E. Vena, Paul Arora, Christine M. Friedenreich, Darren R. Brenner

**Affiliations:** 1 Department of Cancer Epidemiology and Prevention Research, CancerControl Alberta, Alberta Health Services, Calgary, T2S 3C3 AB Canada; 2 Bachelor of Health Sciences, Cumming School of Medicine, University of Calgary, Calgary, T2N 4N1 AB Canada; 3 Alberta’s Tomorrow Project, CancerControl Alberta, Alberta Health Services, Calgary, T2T 0J6 AB Canada; 4 Dalla Lana School of Public Health, University of Toronto, Toronto, M5T 3M7 ON Canada; 5 Departments of Oncology and Community Health Sciences, Cumming School of Medicine, University of Calgary, Calgary, T2N 4Z6 AB Canada

## Abstract

The importance of body size versus weight distribution for cancer risk is unclear. We investigated associations between measures of body size and shape and the risk of developing cancer. The study population consisted of 26,607 participants from the Alberta’s Tomorrow Project cohort. Two main measures of body shape and size were examined: i) body mass index (BMI) and ii) waist circumference (WC). Incident cancers were identified via linkage to the Alberta Cancer Registry. Cox proportional hazards models were used. Males and females classified as obese (BMI ≥ 30 kg /m^−2^) have a 33% and 22% increased risk of all-cancer, respectively, than their normal weight counterparts. Similar all-cancer risk increases are observed for those above sex-specific WC guidelines. Mutual adjustment for WC attenuates the association between BMI and all-cancer risk, especially among females. Central adiposity appears to be a stronger predictor of all-cancer risk than body size.

## Abstract (web summary)

Obesity is linked to increased cancer risk but the impact of body size versus weight distribution in determining the increased risk is unclear. Here the authors examined body mass index, waist circumference, and waist to hip ratio in relation to all-cancer incidence and incidence of seven individual cancers in a population of approximately 26,000 individual and conclude that central adiposity appears to be a stronger predictor of all-cancer risk than body size.

## Introduction

In 2014 national surveys, 61.8% of Canadian men and 46.2% of Canadian women were classified as overweight (body mass index [BMI] ≥ 25 kg/m^2^) or obese (BMI ≥ 30 kg/m^2^) based on self-reported height and weight^1^. The prevalence of obesity in Canada began to rise at a steady rate in the 1980s and recent projections suggest that by 2019, overweight and obese adults will outnumber normal-weight adults in half of the Canadian provinces^2^.

Excess body weight is a strong risk factor for several chronic diseases including Type II diabetes, cardiovascular disease, and certain types of cancer^3^. To date, reports by the World Cancer Research Fund (WCRF) and American Institute for Cancer Research (AICR) conclude that excess body fatness increases the risk of 12 site-specific cancers including: esophageal^4^, pancreatic^5^, colorectal^6^, breast (postmenopausal)^7^, endometrial^8^, kidney^9^, gallbladder^10^, stomach^11^, liver^12^, ovarian^13^, advanced prostate cancers^14^, and mouth, pharynx and larynx cancers^15^.

The WCRF estimates that ~21% of these 11 types of cancer could be prevented each year in the United States if the entire adult population maintained a healthy body weight (BMI < 25 kg/m^2^)^16^. In 2012, we estimated in Alberta, Canada the proportion of cancer cases attributable to excess body weight and found positive associations of overweight/obesity with risk of seven cancer sites (colorectal, breast, endometrial, esophageal, gallbladder, pancreatic, and kidney cancers); overall, we concluded that 17% and 12% cancers among males and females, respectively, could be attributed to excess body weight^17^.

While a large evidence base on excess body weight and body size and cancer risk has accumulated, some nuances of these associations still warrant further investigation. The key remaining etiologic question is whether weight distribution or total body size is more important for cancer risk. For example, a recent systematic review and meta-analysis by Aune et al. (2015) examining anthropometric factors and endometrial cancer risk, as part of the WCRF Continuous Update Project, noted that a limited number of studies reported on waist circumference (WC) (4 out of 30) and waist-to-hip ratio (WHR) (5 out of 30), and few studies performed mutual adjustments for BMI and WC to elucidate their independent role in endometrial cancer risk^18^. It is plausible that measures of body fat distribution, such as WC and WHR could be stronger determinants of cancer risk than overall body size, since WC and WHR have been shown to be better predictors of morbidity and all-cause mortality than BMI^19–21^.

To our knowledge, few Canadian prospective cohort studies have examined the association between body size and shape and cancer risk. In the present study, we utilized data from Alberta’s Tomorrow Project (ATP), a prospective cohort study that included three measures of body size and shape available for participants enrolled at baseline: (i) BMI, (ii) WC, and (iii) WHR. Analyses using waist-to-height ratio (WtHR) were also conducted. Our two main objectives were: (i) to examine the associations between various measures of body shape and size and the risk of all-cancer and site-specific cancers, and; (ii) to examine which is the stronger predictor of cancer risk—total body mass or central body fatness.

## Results

### Overview of study population

The analytic study population consisted of 26,607 participants who completed the HLQ, CDHQ, and PYTPAQ, consented to data linkage, and had no previous cancer diagnosis in the ACR. This sample size resulted in 328,095 person-years of follow-up. Of these, a total of 2370 participants (1012 males and 1358 females) had an incident, primary cancer identified by the ACR during the follow-up period (mean of 7.2 years for cancer cases and 12.8 years for non-cancer cases).

Table 1 presents cancer status and sex by body size and shape, socio-demographic, and lifestyle variables. While the proportion of overweight category was similar between cancer cases and non-cancer cases, the proportions of participants in the obese category were higher among cancer cases than non-cases: 33.5% vs. 26.9% for males, and 32.1% vs. 25.5% for females. Mean WC and WHR were slightly higher among cancer cases than non-cancer cases. Males and females who did not develop cancer tended to be younger, never smokers, premenopausal (females), and reported higher levels of education, total-household income, and mean total physical activity at baseline compared to their male and female counterparts who developed cancer. Other characteristics appeared to be similar between cancer cases and non-cancer cases.

**Table 1:** Descriptive statistics for consenting ATP participants (*n* = 26,607)

	Males		Females	
	Cancer	Non-cancer	Cancer	Non-cancer
	(*n* = 1012)	(*n* = 9014)	(*n* = 1358)	(*n* = 15,223)
Follow-up time (years)	7.1 (4.0)	13.0 (2.5)	7.2 (4.0)	12.7 (2.5)
Age (years)	57.3 (8.2)	50.3 (9.0)	55.6 (9.1)	50.5 (9.1)
BMI (kg/m^2^)	28.8 (4.8)	28.0 (4.4)	28.3 (6.4)	27.2 (5.9)
Underweight (<18.5 kg/m^2^)	0.2% (*n* = 2)	0.1% (*n* = 12)	0.7% (*n* = 10)	1.0% (*n* = 157)
Normal weight (18.5 to <25 kg/m^2^)	18.8% (*n* = 190)	23.3% (*n* = 2104)	34.5% (*n* = 469)	40.0% (*n* = 6092)
Overweight (25 to <30 kg/m^2^)	47.3% (*n* = 479)	49.4% (*n* = 4454)	32.3% (*n* = 438)	33.3% (*n* = 5,072)
Obese Class I (30 to <35 kg/m^2^)	22.9% (*n* = 232)	20.2% (*n* = 1821)	18.0% (*n* = 244)	15.6% (*n* = 2371)
Obese Class II (35 to <40 kg/m^2^)	8.1% (*n* = 82)	5.1% (*n* = 460)	8.6% (*n* = 117)	6.2.% (*n* = 936)
Obese Class III (≥40 kg/m^2^)	2.5% (*n* = 25)	1.6% (*n* = 142)	5.5% (*n* = 74)	3.7% (*n* = 558)
Waist circumference (cm)	103.8 (13.5)	100.5 (12.4)	91.9 (16.3)	88.2 (14.8)
Below guidelines^a^	49.3% (*n* = 499)	60.5% (*n* = 5450)	47.6% (*n* = 647)	56.6% (*n* = 8615)
Above guidelines^b^	50.4% (*n* = 510)	39.2% (*n* = 3531)	51.5% (*n* = 699)	42.9% (*n* = 6537)
Waist-to-hip ratio	0.99 (0.07)	0.97 (0.07)	0.85 (0.07)	0.83 (0.07)
Below guidelines^c^	9.5% (*n* = 96)	14.9% (*n* = 1344)	53.2% (*n* = 723)	61.6% (*n* = 9381)
Above guidelines^d^	89.4% (*n* = 905)	83.7% (*n* = 7544)	45.6% (*n* = 619)	37.6% (*n* = 5730)
Mean total physical activity (MET-h/week)	151.8 (74.3)	173.8 (74.9)	145.6 (68.7)	157.3 (65.0)
Mean daily energy intake (kcal)	2156.7 (995.1)	2245.2 (1025.0)	1595.5 (647.2)	1642.8 (668.0)
Mean daily fiber intake (g)	20.2 (9.3)	20.7 (9.8)	17.7 (8.2)	18.4 (8.8)
Mean daily alcohol intake (g)	17.5 (41.3)	16.7 (45.5)	5.9 (13.8)	6.6 (19.8)
Marital status				
Married or living with someone	84.4% (*n* = 854)	83.2% (*n* = 7497)	72.4% (*n* = 982)	76.4% (*n* = 11,623)
Divorced, separated or widowed	10.6% (*n* = 107)	10.3% (*n* = 926)	21.7% (*n* = 295)	18.4% (*n* = 2796)
Single	5.0% (*n* = 51)	6.6% (*n* = 590)	5.9% (*n* = 80)	5.3% (*n* = 803)
Education				
High school or less	32.8% (*n* = 332)	24.2% (*n* = 2178)	34.9% (*n* = 474)	29.5% (*n* = 4487)
Some post-high school	21.9% (*n* = 222)	21.3% (*n* = 1920)	25.8% (*n* = 350)	24.3% (*n* = 3700)
Post high school certificate or degree	45.3% (*n* = 458)	54.5% (*n* = 4915)	39.3% (*n* = 534)	46.2% (*n* = 7035)
Total household income				
$0–$49,999	32.4% (*n* = 328)	23.0% (*n* = 2070)	45.1% (*n* = 613)	34.3% (*n* = 5217)
$50,000–$99,999	42.8% (*n* = 433)	44.5% (*n* = 4008)	36.5% (*n* = 496)	39.1% (*n* = 5947)
≥$100,000	22.9% (*n* = 232)	31.2% (*n* = 2811)	14.8% (*n* = 201)	23.8% (*n* = 3626)
Pack-years of smoking (years)	15.7 (19.0)	10.3 (15.0)	12.5 (16.7)	7.5 (12.2)
Smoking status				
Never	33.4% (*n* = 338)	42.9% (*n* = 3866)	39.7% (*n* = 539)	47.0% (*n* = 7162)
Former	46.9% (*n* = 475)	39.2% (*n* = 3534)	36.8% (*n* = 499)	36.2% (*n* = 5518)
Current	19.7% (*n* = 199)	17.9% (*n* = 1609)	23.5% (*n* = 319)	16.6% (*n* = 2531)
Self-reported history of diabetes				
No	91.9% (*n* = 930)	94.5% (*n* = 8520)	94.5% (*n* = 1284)	96.0% (*n* = 14,610)
Yes	8.0% (*n* = 81)	5.4% (*n* = 488)	5.5% (*n* = 74)	4.0% (*n* = 604)
Self-reported family history of cancer				
No	41.9% (*n* = 424)	50.0% (*n* = 4506)	38.6% (*n* = 524)	45.9% (*n* = 6983)
Yes	58.1% (*n* = 588)	50.0% (*n* = 4508)	61.4% (*n* = 834)	54.1% (*n* = 8240)
Menopausal status				
Pre-menopausal	N/A	N/A	31.8% (*n* = 432)	52.6% (*n* = 8015)
Post-menopausal	N/A	N/A	68.2% (*n* = 962)	47.4% (*n* = 7207)
Years of birth control use				
0–5 years	N/A	N/A	66.3% (*n* = 900)	57.6% (*n* = 8796)
>5 years	N/A	N/A	33.6% (*n* = 456)	42.1% (*n* = 6416)

Note: Means and standard deviations are reported for continuous variables and proportions and sample sizes are reported for categorical variables

^a^Waist circumference below guidelines:   <102  cm for men,   <88  cm for women

^b^Waist circumference above guidelines:  ≥ 102  cm for men,  ≥ 88 cm for women

^c^Waist-to-hip ratio below guidelines:   <0.9  cm for men,   <0.85  cm for women

^d^Waist-to-hip ratio above guidelines:  ≥ 0.9  cm for men,  ≥ 0.85  cm for women

### Overall body size and cancer risk

The sex-specific results of BMI categories and all- and site-specific cancer incidence are presented in Table 2. Information on BMI was available for 26,541 participants (10,003 males and 16,538 females) who met other inclusion criteria; however, after participants classified as underweight (BMI < 18.5 kg/m^2^) were excluded, 26,360 participants (9989 males and 16,371 females) were included in these analyses. For males, positive trends with increasing BMI were observed for the incidence of all-cancer (*P*_trend_ ≤ 0.001, Cox proportional hazard), colon cancer (*P*_trend_ ≤ 0.001, Cox proportional hazard), non-Hodgkin lymphoma (*P*_trend_ ≤ 0.01, Cox proportional hazard), and hematological cancers (*P*_trend_ = 0.04, Cox proportional hazard). Compared to males of normal weight, males with a BMI ≥ 30 kg/m^2^ had a 33% increased risk of all-cancer during the follow-up period (HR = 1.33, 95% CI: 1.10, 1.60). Males with a BMI ≥ 30 kg/m^2^ had 2.71 (95% CI: 1.28, 5.70) and 2.47 (95% CI: 1.10, 5.57) times the risk of colon cancer and non-Hodgkin lymphoma, respectively. No significant associations between BMI and the risk of prostate cancer, lung cancer, or leukemia were observed among males. For females, positive trends with increasing BMI were observed for all-cancers (*P*_trend_ ≤ 0.01, Cox proportional hazard) and endometrial cancer (*P*_trend_ ≤ 0.001, Cox proportional hazard). A significant inverse association between BMI and lung cancer was observed among females (*P*_trend_ = 0.03, Cox proportional hazard); although this inverse trend was not significant among males, it was observed to be in the same direction. Higher BMI was also associated with a reduced risk of breast cancer among premenopausal women (*P*_trend_ = 0.03, Cox proportional hazard). Compared to females of normal weight, females with a BMI ≥ 30 kg/m^2^ had a 22% increased risk of all-cancer during the follow-up period (HR = 1.22, 95% CI: 1.06, 1.40, *P* < 0.001). A strong positive association between a BMI ≥ 30 kg/m^2^ and the risk of endometrial cancer was observed (HR = 4.52, 95% CI: 2.69, 7.61). Overweight (BMI ≥ 25 kg/m^2^), but not obese, females had a significantly higher risk of colon cancer (HR = 1.80, 95% CI: 1.11, 2.96). No significant associations between BMI and the incidence of postmenopausal breast cancer, colon cancer, leukemia, non-Hodgkin lymphoma, and hematological cancers were observed among females. The aforementioned sex-specific findings for BMI and cancer risk were robust to sensitivity analyses whereby cancers occurring < 2 years following baseline data collection were removed with one exception; the significant inverse trend between higher BMI and the risk of premenopausal breast cancer observed in the multivariate-adjusted model (*P*_trend_ = 0.03, Cox proportional hazard) was not significant in the latency multivariate-adjusted model (*P*_trend_ = 0.14, Cox proportional hazard) (Supplementary Table 1).

**Table 2:** Results from analyses of BMI categories^a^ and cancer incidence among ATP participants with a BMI ≥ 18.5 kg/m^2^ (*n* = 26,360)

	Cases (male)	Age-adjusted (male)	Cases (male)	Multivariate-adjusted^b^ (male)	Cases (female)	Age-adjusted (female)	Cases (female)	Multivariate-adjusted^b^ (female)
All-cancer								
Normal	190	1.0 (Ref)	185	1.0 (Ref)	469	1.0 (Ref)	447	1.0 (Ref)
Overweight	479	1.07 (0.92, 1.27)	471	1.08 (0.91, 1.29)	438	0.96 (0.84, 1.10)	418	0.95 (0.83, 1.09)
Obese	339	1.32** (1.12, 1.58)	331	1.33** (1.10, 1.60)	435	1.21** (1.06, 1.38)	420	1.22** (1.06, 1.40)
* P for trend*		<0.001		<0.001		<0.01		<0.01
Prostate cancer								
Normal	74	1.0 (Ref)	72	1.0 (Ref)	–	–	–	–
Overweight	207	1.18 (0.91, 1.54)	205	1.17 (0.89, 1.53)	–	–	–	–
Obese	119	1.18 (0.88, 1.58)	115	1.17 (0.87, 1.58)	–	–	–	–
* P for trend*		0.31		0.34		–		–
Breast cancer—premenopausal								
Normal	–	–	–	–	101	1.0 (Ref)	98	1.0 (Ref)
Overweight	–	–	–	–	53	0.77 (0.55, 1.07)	50	0.74^†^ (0.52, 1.06)
Obese	–	–	–	–	37	0.78 (0.53, 1.31)	34	0.67^†^ (0.44, 1.01)
* P for trend*		–		–		0.12		0.03
Breast cancer—postmenopausal								
Normal	–	–	–	–	90	1.0 (Ref)	85	1.0 (Ref)
Overweight	–	–	–	–	92	0.89 (0.66, 1.18)	89	0.92 95 (0.70, 1.28)
Obese	–	–	–	–	104	1.21 (0.91, 1.60)	99	1.28 (0.95, 1.74)
* P for trend*		–		–		0.18		0.11
Endometrial cancer								
Normal	–	–	–	–	23	1.0 (Ref)	20	1.0 (Ref)
Overweight	–	–	–	–	31	1.48 (0.86, 2.54)	26	1.48 (0.82, 2.67)
Obese	–	–	–	–	70	4.27** (2.66, 6.87)	65	4.52** (2.69, 7.61)
* P for trend*		–		–		<0.001		<0.001
Colon cancer								
Normal	9	1.0 (Ref)	9	1.0 (Ref)	26	1.0 (Ref)	26	1.0 (Ref)
Overweight	38	1.80 (0.87, 3.72)	37	1.66 (0.79, 3.45)	49	1.88** (1.16, 3.03)	48	1.80* (1.11, 2.92)
Obese	36	2.97** (1.43, 6.17)	36	2.71** (1.28, 5.70)	32	1.55^†^ (0.92, 2.62)	32	1.49 (0.87, 2.56)
* P for trend*		<0.001		<0.001		0.10		0.14
Lung cancer								
Normal	19	1.0 (Ref)	18	1.0 (Ref)	54	1.0 (Ref)	51	1.0 (Ref)
Overweight	34	0.73 (0.42, 1.28)	32	0.78 (0.43, 1.41)	50	0.82 (0.56, 1.21)	46	0.80 (0.53, 1.19)
Obese	19	0.71 (0.38, 1.34)	19	0.71 (0.37, 1.39)	28	0.58* (0.36, 0.91)	28	0.59* (0.36, 0.95)
* P for trend*		0.31		0.33		0.02		0.03
Leukemia								
Normal	12	1.0 (Ref)	12	1.0 (Ref)	18	1.0 (Ref)	17	1.0 (Ref)
Overweight	23	0.84 (0.42, 1.69)	23	0.88 (0.43, 1.78)	14	0.79 (0.39, 1.59)	14	0.76 (0.37, 1.55)
Obese	19	1.23 (0.59, 2.53)	17	1.15 (0.54, 2.46)	12	0.86 (0.41, 1.79)	11	0.68 (0.31, 1.52)
* P for trend*		0.49		0.67		0.64		0.33
Non-Hodgkin lymphoma								
Normal	8	1.0 (Ref)	8	1.0 (Ref)	14	1.0 (Ref)	13	1.0 (Ref)
Overweight	19	1.05 (0.46, 2.396)	19	1.08 (0.47, 2.49)	17	1.21 (0.59, 2.47)	16	1.20 (0.57, 2.50)
Obese	26	2.52* (1.14, 5.57)	26	2.47* (1.10, 5.57)	16	1.44 (0.70, 2.98)	16	1.46 (0.69, 3.11)
* P for trend*		0.01		0.01		0.32		0.32
Hematological cancers								
Normal	21	1.0 (Ref)	21	1.0 (Ref)	33	1.0 (Ref)	31	1.0 (Ref)
Overweight	43	0.90 (0.54, 1.52)	43	0.93 (0.55, 1.58)	32	0.98 (0.60, 1.60)	31	0.96 (0.58, 1.59)
Obese	45	1.67* (0.99, 2.80)	43	1.61^†^ (0.94, 2.75)	29	1.13 (0.68, 1.86)	28	1.05 (0.62, 1.79)
* P for trend*		0.02		0.04		0.66		0.86

Note: Hazard ratios (HRs) and 95% confidence intervals (CIs) from Cox regression models are presented

^a^Normal = ≥18.5 to <25 kg/m^2^; overweight = ≥25 to <30 kg/m^2^; obese = ≥30 kg/m^2^. Those with a BMI < 18.5  kg/m^2^ were excluded from these analyses

^b^Adjusted for: age (continuous), ethnicity (white/other), marital status (married or living with someone/divorced, separated, or widowed/single, never married), highest level of education (high school or less/some post-high school education/post-high school certificate or degree), total household income ($0–$49,999/$50,000–$99,999/≥$100,000), geographical area of residence (urban/rural), smoking status (current/former/never), alcohol consumption (grams of ethanol per day), energy intake (kilocalories per day), total physical activity (MET-hours per week), history of diabetes (yes/no), family history of cancer (yes/no), pack-years of cigarettes (lung cancer only), fiber intake (grams per day) (colon cancer only), menopausal status (pre-menopause/post-menopause) (endometrial cancers), years of birth control use (0–5 years/ > 5 years), (breast and endometrial cancers), history of breast cancer screening (yes/no) (breast cancer only), history of colon cancer screening (yes/no) (colon cancer only), history of prostate cancer screening (yes/no) (prostate cancer only), and ever used female hormones for menopause (yes/no) (breast cancer only)

**p < 0.01; *p < 0.05; ^†^*p* < 0.1

### WC attenuates the effects of body size

Results were no longer statistically significant for BMI and all-cancer risk when adjusting for all covariates, as well as WC as a dichotomous variable (HR_males_ = 1.20, 95% CI: 0.94, 1.52 and HR_females_ = 1.11, 95% CI: 0.90, 1.35) (Supplementary Table 2). Males with a BMI ≥ 30 kg/m^2^ had a marginally increased risk of colon cancer (HR = 2.28, 95% CI: 0.92, 5.67) and a reduced risk of lung cancer (HR = 0.36, 95% CI: 0.14, 0.90), in models that adjusted for all covariates as well as WC. For females, a BMI ≥ 30 kg/m^2^ was associated with an increased risk of endometrial cancer (HR = 3.12, 95% CI: 1.43, 6.81); however, this risk was attenuated compared to results where WC was not adjusted for. Further risk reduction was also observed between increasing BMI and premenopausal breast cancer when adjusting for all covariates and WC (*P*_trend_ = 0.02, Cox proportional hazard).

Table 3 presents sex-specific results of WC cutoff categories and all- and site-specific cancer incidence. Information on WC was available for 26,488 individuals (*n* = 9990 males and 16,498 females) who met other inclusion criteria. As compared with males who had a WC below guidelines, males with a WC above guidelines (≥102 cm) had a significantly elevated risk of all-cancer (HR = 1.22, 95% CI: 1.07, 1.38), as well as of three other site-specific cancers (colon, non-Hodgkin lymphoma, and hematological), which ranged from 1.56 (95% CI: 1.05, 2.30) for hematological cancers to 2.11 (95% CI: 1.19, 3.75) for non-Hodgkin lymphoma. Females with a WC above guidelines (≥88 cm) had a significantly elevated risk of all-cancer compared to females with a WC below guidelines (HR = 1.17, 95% CI: 1.04, 1.31), as well as endometrial cancer (HR = 3.14, 95% CI: 2.04, 4.85), but no other site-specific associations were observed. Sex-specific findings for WC and cancer risk were not markedly different when cancers occurring <2 years after baseline data collection were removed (Supplementary Table 3).

**Table 3:** Results from analyses of waist circumference^a,b^ and cancer incidence (*n* = 26,488)

	Cases (male)	Age-adjusted (male)	Cases (male)	Multivariate-adjusted^c^ (male)	Cases (female)	Age-adjusted (female)	Cases (female)	Multivariate-adjusted^c^ (female)
All-cancer								
Below	499	1.0 (Ref)	488	1.0 (Ref)	647	1.0 (Ref)	620	1.0 (Ref)
Above	510	1.24** (1.10, 1.41)	499	1.22** (1.07, 1.38)	699	1.19** (1.07, 1.33)	671	1.17** (1.04, 1.31)
Prostate cancer								
Below	206	1.0 (Ref)	202	1.0 (Ref)	–	–	–	–
Above	194	1.12 (0.92, 1.37)	190	1.14 (0.93, 1.39)	–	–	–	–
Breast cancer—premenopausal								
Below	–	–	–	–	124	1.0 (Ref)	121	1.0 (Ref)
Above	–	–	–	–	70	0.98 (0.73, 1.32)	64	0.87 (0.63, 1.21)
Breast cancer—postmenopausal								
Below	–	–	–	–	125	1.0 (Ref)	120	1.0 (Ref)
Above	–	–	–	–	161	1.16 (0.92, 1.47)	154	1.20 (0.94, 1.54)
Endometrial cancer								
Below	–	–	–	–	34	1.0 (Ref)	30	1.0 (Ref)
Above	–	–	–	–	88	3.07** (2.06, 4.59)	81	3.14** (2.04, 4.85)
Colon cancer								
Below	32	1.0 (Ref)	32	1.0 (Ref)	49	1.0 (Ref)	48	1.0 (Ref)
Above	50	1.89** (1.21, 2.96)	49	1.67* (1.06, 2.65)	59	1.28 (0.87, 1.87)	59	1.25 (0.84, 1.86)
Lung cancer								
Below	32	1.0 (Ref)	30	1.0 (Ref)	72	1.0 (Ref)	67	1.0 (Ref)
Above	42	1.49^†^ (0.94, 2.36)	41	1.20 (0.73, 1.96)	64	0.83 (0.59, 1.16)	62	0.74 (0.51, 1.06)
Leukemia								
Below	27	1.0 (Ref)	27	1.0 (Ref)	21	1.0 (Ref)	20	1.0 (Ref)
Above	27	1.29 (0.75, 2.21)	25	1.25 (0.71, 2.18)	23	0.79 (0.39, 1.59)	22	1.08 (0.57, 2.03)
Non-Hodgkin lymphoma								
Below	20	1.0 (Ref)	20	1.0 (Ref)	23	1.0 (Ref)	22	1.0 (Ref)
Above	33	2.18** (1.24, 3.81)	33	2.11** (1.19, 3.75)	24	1.10 (0.62, 1.97)	23	1.05 (0.57, 1.92)
Hematological cancers								
Below	49	1.0 (Ref)	49	1.0 (Ref)	45	1.0 (Ref)	43	1.0 (Ref)
Above	60	1.61* (1.10, 2.35)	58	1.56* (1.05, 2.30)	49	1.17 (0.78, 1.77)	47	1.12 (0.73, 1.71)

Note: Hazard ratios (HRs) and 95% confidence intervals (CIs) from Cox regression models are presented

^a^Waist circumference below guidelines: <102 cm for men, <88 cm for women

^b^Waist circumference above guidelines: ≥102 cm for men, ≥88 cm for women

^c^Adjusted for: age (continuous), ethnicity (white/other), marital status (married or living with someone/divorced, separated, or widowed/single, never married), highest level of education (high school or less/some post-high school education/post-high school certificate or degree), total household income ($0 to $49,999/$50,000 to $99,999/≥$100,000), geographical area of residence (urban/rural), smoking status (current/former/never), alcohol consumption (grams of ethanol per day), energy intake (kilocalories per day), total physical activity (MET-hours per week), history of diabetes (yes/no), family history of cancer (yes/no), pack-years of cigarettes (lung cancer only), fiber intake (grams per day) (colon cancer only), menopausal status (pre-menopause/post-menopause) (endometrial cancer), years of birth control use (0–5 years/>5 years), (breast and endometrial cancers), history of breast cancer screening (yes/no) (breast cancer only), history of colon cancer screening (yes/no) (colon cancer only), history of prostate cancer screening (yes/no) (prostate cancer only), and ever used female hormones for menopause (yes/no) (breast cancer only)

**p < 0.01; *p < 0.05; ^†^*p* < 0.1

Statistically significant results for sex-specific findings for WC and cancer risk were also similar when adjusting for all covariates, as well as dichotomous BMI (Supplementary Table 4). Males with a WC above guidelines still had an increased risk for all-cancer (HR = 1.20, 95% CI: 1.04, 1.39), colon cancer (HR = 1.67, 95% CI: 1.06, 2.65), non-Hodgkin lymphoma (HR = 2.10, 95% CI: 1.09, 4.04), and hematological cancers (HR = 1.63, 95% CI: 1.04, 2.55) in models that adjusted for all covariates and BMI. Females reporting a WC above guidelines, also had an increased risk of all-cancer (HR = 1.21, 95% CI: 1.04, 1.40) and endometrial cancer (HR = 2.41, 95% CI: 1.38, 4.22) in models that adjusted for all covariates and BMI.

We further examined WC in quartiles (Supplementary Table 5). Females in the highest quartile for WC had a significantly increased all-cancer risk (HR_Q4vsQ1_ = 1.34, 95% CI: 1.14, 1.58) in the multivariate-adjusted model. This risk was slightly attenuated in a final model additionally adjusted for BMI (HR_Q4vsQ1_ = 1.26, 95% CI: 0.97, 1.64). Females in the highest and second highest WC quartiles were at an increased risk for endometrial cancer (HR_Q4vsQ1_ = 4.48, 95% CI: 2.63, 7.63; HR_Q3vsQ1_ = 2.34, 95% CI: 1.32, 4.13).

**Fig. 1:**
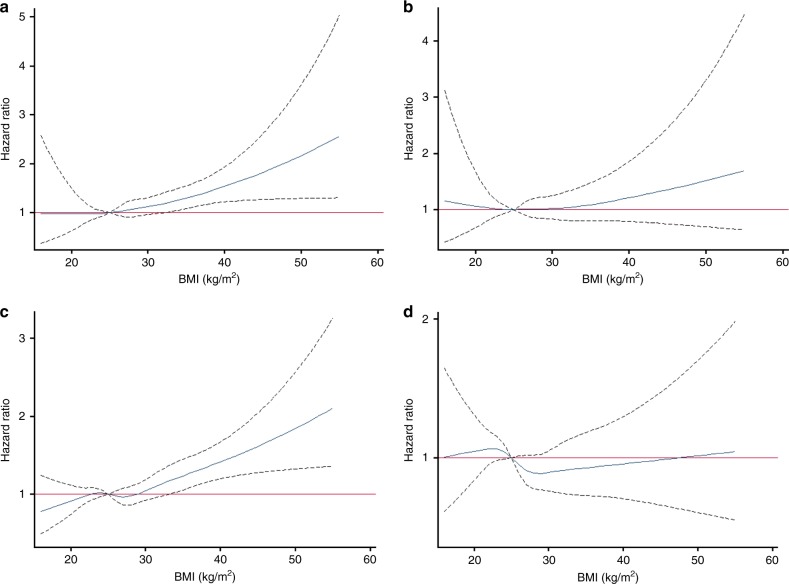
**a** Effect of BMI on all-cancer risk among males (989 cancer cases) in the Alberta’s Tomorrow Project (*N* = 9790) adjusted for covariates. **b** Effect of BMI on all-cancer risk among males (986 cancer cases) in the Alberta’s Tomorrow Project (*N* = 9761) adjusted for covariates and continuous waist circumference. **c** Effect of BMI on all-cancer risk among females (1292 cancer cases) in the Alberta’s Tomorrow Project (*N* = 15,945) adjusted for covariates. **d** Effect of BMI on all-cancer risk among females (1287 cancer cases) in the Alberta’s Tomorrow Project (*N* = 15,886) adjusted for covariates and continuous waist circumference. For ease of presentation, those with a BMI > 55 kg/m^2^ were excluded. All models were adjusted for age (continuous), ethnicity (white/other), marital status (married or living with someone/divorced, separated, or widowed/single, never married), highest level of education (high school or less/some post-high school education/post-high school certificate or degree), total household income ($0–$49,999/$50,000 to $99,999/≥$100,000), geographical area of residence (urban/rural), smoking status (current/former/never), alcohol consumption (grams of ethanol per day), energy intake (kilocalories per day), total physical activity (MET-hours per week), history of diabetes (yes/no), and family history of cancer (yes/no). This figure contains the hazard ratio represented by the blue line and the 95% confidence intervals represented by the dashed black lines on either side of the blue line. The red line represents the null value of 1 for the hazard ratio

We generated multiple dose–response curves to provide a visual presentation of how adjustment for WC attenuates the effects of body size. The sex-specific results of the association between continuous BMI and all-cancer risk, before and after adjustment for continuous WC are displayed in Fig. 1. For males, a significant positive association between BMI and all-cancer risk is apparent at ~30 kg/m^2^ (Fig. 1a); however, this association is no longer statistically significant following adjustment for WC (Fig. 1b). Similarly, for females, a statistically significant positive association between BMI and all-cancer risk arises at ~35 kg/m^2^ (Fig. 1c), which disappears after adjustment for WC (Fig. 1d).

**Fig. 2:**
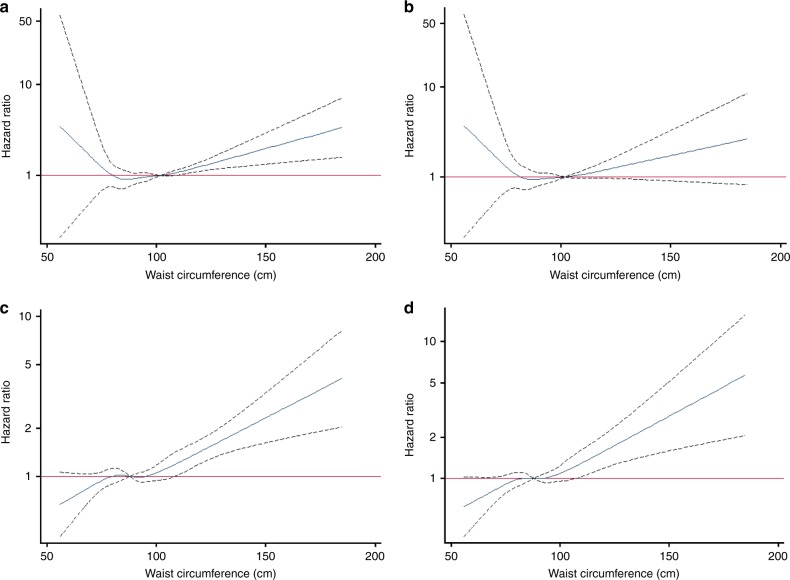
**a** Effect of waist circumference on all-cancer risk among males (987 cancer cases) in the Alberta’s Tomorrow Project (*N* = 9780) adjusted for covariates. **b** Effect of waist circumference on all-cancer risk among males (986 cancer cases) in the Alberta’s Tomorrow Project (*N* = 9763) adjusted for covariates and continuous BMI. **c** Effect of waist circumference on all-cancer risk among females (1291 cancer cases) in the Alberta’s Tomorrow Project (*N* = 15,935) adjusted for covariates. **d** Effect of waist circumference on all-cancer risk among females (1289 cancer cases) in the Alberta’s Tomorrow Project (*N* = 15,911) adjusted for covariates and continuous BMI. All models were adjusted for age (continuous), ethnicity (white/other), marital status (married or living with someone/divorced, separated, or widowed/single, never married), highest level of education (high school or less/some post-high school education/post-high school certificate or degree), total household income ($0–$49,999/$50,000 to $99,999/≥$100,000), geographical area of residence (urban/rural), smoking status (current/former/never), alcohol consumption (grams of ethanol per day), energy intake (kilocalories per day), total physical activity (MET-hours per week), history of diabetes (yes/no) and family history of cancer (yes/no). This figure contains the hazard ratio represented by the blue line and the 95% confidence intervals represented by the dashed black lines on either side of the blue line. The red line represents the null value of 1 for the hazard ratio

Figure 2 displays the sex-specific results of the association between continuous WC and all-cancer risk, before and after adjustment for continuous BMI. Beyond the reference value of 102 cm for males, there is a significant positive association between WC and the risk of all-cancer (Fig. 2a), an effect which appears to remain following adjustment for BMI though blunted (Fig. 2b). Beyond the reference value of 88 cm for females, there is a positive association between WC and the risk of all-cancer, with a steeper increase in risk at higher values of WC (Fig. 2c), which is virtually unchanged after adjustment for BMI (Fig. 2d).

### Other measures of body shape (WHR and WtHR)

The sex-specific results of WHR quartiles and all- and site-specific cancer incidence are presented in Table 4. Information on WHR was available for 26,342 individuals (*n* = 9889 males and 16,453 females) who met other inclusion criteria. For males, significant positive trends with increasing quartiles of WHR were observed for the incidence of all-cancer (*P*_trend_ = 0.01, Cox proportional hazard), prostate cancer (*P*_trend_ ≤ 0.01, Cox proportional hazard), and a marginally statistically significant positive trend was observed for colon cancer (*P*_trend_ = 0.06, Cox proportional hazard). Males in the highest quartile versus the lowest quartile of WHR had a 28% (95% CI: 1.06, 1.56) increased risk of all-cancer and 42% (95% CI: 1.05, 1.91) increased risk of prostate cancer during the follow-up period. Males in the second quartile versus the lowest quartiles of WHR had a significantly higher risk of non-Hodgkin lymphoma (HR_Q2vsQ1_ = 4.20, 95% CI: 1.42, 12.39). For females, statistically significant positive trends with increasing quartiles of WHR were observed for the incidence of all-cancer (*P*_trend_ = 0.05, Cox proportional hazard) and endometrial cancer (*P*_trend_ ≤ 0.01, Cox proportional hazard). Females in the highest quartile versus the lowest quartile of WHR had a 19% (95% CI: 1.01, 1.41) increased risk of all-cancer during the follow-up period. An increased risk of endometrial cancer was observed for females in both the highest and second highest quartiles of WHR (HR_Q4vsQ1_ = 1.97, 95% CI: 1.09, 3.57; HR_Q3vsQ1_ = 2.10, 95% CI: 1.17, 3.75). When we examined sex-specific findings for quartiles of WHR and cancer risk in sensitivity analyses excluding cancers that occurred < 2 years after baseline data collection, effects estimates were not meaningfully altered (Supplementary Table 6).

**Table 4:** Results from analyses of waist-to-hip ratio (quartiles)^a^ and cancer incidence (*n* = 26,342)

	Cases (male)	Age-adjusted (male)	Cases (male)	Multivariate-adjusted^b^ (male)	Cases (female)	Age-adjusted (female)	Cases (female)	Multivariate-adjusted^b^ (female)
All-cancer								
1st Quartile	172	1.0 (Ref)	168	1.0 (Ref)	263	1.0 (Ref)	254	1.0 (Ref)
2nd Quartile	231	1.13 (0.93, 1.38)	225	1.11 (0.91, 1.36)	327	1.16† (0.99, 1.37)	315	1.15 (0.97, 1.35)
3rd Quartile	269	1.16 (0.95, 1.40)	267	1.15 (0.94, 1.39)	345	1.18* (1.00, 1.38)	330	1.15 (0.97, 1.35)
4th Quartile	329	1.34** (1.11, 1.61)	319	1.28* (1.06, 1.56)	407	1.28** (1.09, 1.50)	388	1.19* (1.01, 1.41)
* P for trend*		<0.01		0.01		<0.01		0.05
Prostate cancer								
1st Quartile	70	1.0 (Ref)	68	1.0 (Ref)	–	–	–	–
2nd Quartile	82	0.98 (0.71, 1.35)	82	1.0 (0.72, 1.38)	–	–	–	–
3rd Quartile	107	1.11 (0.82, 1.50)	105	1.11 (0.82, 1.51)	–	–	–	–
4th Quartile	139	1.36* (1.01, 1.81)	135	1.42* (1.05, 1.91)	–	–	–	–
* P for trend*		0.02		<0.01		–		–
Breast cancer—premenopausal								
1st Quartile	–	–	–	–	51	1.0 (Ref)	48	1.0 (Ref)
2nd Quartile	–	–	–	–	62	1.36 (0.94, 1.98)	59	1.42^†^ (0.96, 2.10)
3rd Quartile	–	–	–	–	51	1.28 (0.87, 1.89)	50	1.25 (0.83, 1.90)
4th Quartile	–	–	–	–	30	0.90 (0.57, 1.42)	28	0.85 (0.52, 1.39)
* P for trend*		–		–		0.84		0.63
Breast cancer—postmenopausal								
1st Quartile	–	–	–	–	54	1.0 (Ref)	51	1.0 (Ref)
2nd Quartile	–	–	–	–	76	1.17 (0.83, 1.66)	73	1.24 (0.86, 1.77)
3rd Quartile	–	–	–	–	62	0.85 (0.59, 1.22)	61	0.91 (0.63, 1.33)
4th Quartile	–	–	–	–	94	1.09 (0.78, 1.52)	89	1.13 (0.79, 1.62)
* P for trend*						0.95		0.91
Endometrial cancer								
1st Quartile	–	–	–	–	17	1.0 (Ref)	17	1.0 (Ref)
2nd Quartile	–	–	–	–	22	1.24 (0.66, 2.33)	19	1.10 (0.57, 2.13)
3rd Quartile	–	–	–	–	39	2.16** (1.22, 3.82)	36	2.10* (1.17, 3.75)
4th Quartile	–	–	–	–	43	2.26** (1.28, 4.00)	38	1.97* (1.09, 3.57)
* P for trend*		–		–		<0.01		<0.01
Colon cancer								
1st Quartile	11	1.0 (Ref)	11	1.0 (Ref)	21	1.0 (Ref)	21	1.0 (Ref)
2nd Quartile	15	1.15 (0.53, 2.51)	15	1.08 (0.49, 2.36)	26	1.14 (0.64, 2.02)	25	1.08 (0.60, 1.93)
3rd Quartile	23	1.55 (0.75, 3.19)	23	1.40 (0.68, 2.90)	28	1.16 (0.66, 2.05)	28	1.14 (0.64, 2.02)
4th Quartile	33	2.11* (1.06, 4.20)	32	1.77 (0.87, 3.59)	32	1.19 (0.68, 2.08)	32	1.12 (0.63, 1.98)
* P for trend*		0.01		0.06		0.57		0.69
Lung cancer								
1st Quartile	10	1.0 (Ref)	9	1.0 (Ref)	21	1.0 (Ref)	20	1.0 (Ref)
2nd Quartile	18	1.44 (0.67, 3.13)	16	1.48 (0.65, 3.36)	31	1.27 (0.73, 2.21)	30	1.06 (0.60, 1.88)
3rd Quartile	16	1.08 (0.49, 2.38)	16	1.07 (0.47, 2.44)	33	1.22 (0.70, 2.10)	32	0.88 (0.50, 1.55)
4th Quartile	30	1.86^†^ (0.90, 3.82)	30	1.28 (0.59, 2.78)	50	1.53 (0.92, 2.56)	46	0.84 (0.48, 1.45)
* P for trend*		0.12		0.78		0.12		0.36
Leukemia								
1st Quartile	12	1.0 (Ref)	12	1.0 (Ref)	4	1.0 (Ref)	4	1.0 (Ref)
2nd Quartile	12	0.87 (0.39, 1.94)	12	0.88 (0.40, 1.98)	10	2.35 (0.74, 7.50)	10	2.36 (0.74, 7.53)
3rd Quartile	16	1.05 (0.49, 2.23)	16	1.08 (0.50, 2.32)	14	3.18* (1.04, 9.70)	13	2.91^†^ (0.94, 8.99)
4th Quartile	12	0.76 (0.34, 1.71)	10	0.66 (0.28, 1.58)	16	3.35* (1.11, 10.11)	15	2.96^†^ (0.96, 9.15)
* P for trend*		0.63		0.48		0.03		0.07
Non-Hodgkin lymphoma								
1st Quartile	4	1.0 (Ref)	4	1.0 (Ref)	10	1.0 (Ref)	9	1.0 (Ref)
2nd Quartile	19	4.27** (1.45, 12.58)	19	4.20** (1.42, 12.39)	12	1.10 (0.47, 2.54)	12	1.22 (0.51, 2.91)
3rd Quartile	15	3.09* (1.02, 9.38)	15	2.97^†^ (0.97, 9.06)	10	0.87 (0.36, 2.09)	9	0.87 (0.34, 2.21)
4th Quartile	15	3.03* (0.99, 9.21)	15	2.76^†^ (0.90, 8.51)	15	1.16 (0.52, 2.62)	15	1.28 (0.54, 3.00)
* P for trend*		0.24		0.36		0.83		0.74
Hematological								
1st Quartile	17	1.0 (Ref)	17	1.0 (Ref)	15	1.0 (Ref)	14	1.0 (Ref)
2nd Quartile	31	1.62 (0.90, 2.93)	31	1.62 (0.89, 2.94)	23	1.43 (0.74, 2.73)	23	1.56 (0.80, 3.02)
3rd Quartile	32	1.53 (0.84, 2.77)	32	1.52 (0.84, 2.77)	24	1.43 (0.75, 2.73)	22	1.41 (0.72, 2.77)
4th Quartile	27	1.26 (0.68, 2.32)	25	1.14 (0.60, 2.16)	32	1.73† (0.93, 3.23)	31	1.78^†^ (0.93, 3.41)
* P for trend*		0.67		0.90		0.10		0.20

Note: Hazard ratios (HRs) and 95% confidence intervals (CIs) from Cox regression models are presented

^a^Quartiles of WHR for males: 1st Quartile = 0.74–0.92, 2nd Quartile = 0.93–0.97, 3rd Quartile = 0.97–1.02, 4th quartile = 1.02–1.50

Quartiles of WHR for females: 1st Quartile = 0.57–0.78, 2nd Quartile = 0.78–0.82, 3rd Quartile = 0.82–0.87, 4th Quartile = 0.87–1.46

^b^Adjusted for: age (continuous), ethnicity (white/other), marital status (married or living with someone/divorced, separated, or widowed/single, never married), highest level of education (high school or less/some post-high school education/post-high school certificate or degree), total household income ($0–$49,999/$50,000 to $99,999/≥$100,000), geographical area of residence (urban/rural), smoking status (current/former/never), alcohol consumption (grams of ethanol per day), energy intake (kilocalories per day), total physical activity (MET-hours per week), history of diabetes (yes/no), family history of cancer (yes/no), pack-years of cigarettes (lung cancer only), fiber intake (grams per day) (colon cancer only), menopausal status (pre-menopause/post-menopause) (breast and endometrial cancers), years of birth control use (0 to 5 years/> 5 years), (breast and endometrial cancers), history of breast cancer screening (yes/no) (breast cancer only), history of colon cancer screening (yes/no) (colon cancer only), history of prostate cancer screening (yes/no) (prostate cancer only), and ever used female hormones for menopause (yes/no) (breast cancer only)

**p < 0.01; *p < 0.05; ^†^*p* < 0.1

Analyses on sex-specific results of WtHR quartiles for all- and site-specific cancer incidence are presented in Supplementary Table 7. For males, significant positive trends were observed between increasing WtHR and the incidence of all-cancer (*P*_trend_ ≤ 0.01, Cox proportional hazard) and colon cancer (*P*_trend_ = 0.03, Cox proportional hazard) though none of the individual HR estimates for quartiles reached statistical significance. For females, significant positive trends were observed between increasing WtHR and the incidence of all-cancer (*P*_trend_ ≤ 0.01, Cox proportional hazard) and endometrial cancer (*P*_trend_ ≤ 0.01, Cox proportional hazard); women in the highest versus lowest quartile of WtHR had a significantly increased risk of developing all-cancer (HR_Q4vsQ1_ = 1.31, 95% CI: 1.25, 1.53) and endometrial cancer (HR_Q4vsQ1_ = 4.19, 95% CI: 2.42, 7.23) during the follow-up period. In a final model additionally adjusted for BMI, a significantly increased risk of developing endometrial cancer was no longer observed (HR_Q4vsQ1_ = 1.29, 95% CI: 0.47, 3.49).

### Stratified analyses by smoking status

All-cancers and lung cancer for males and females combined were further examined by smoking status for BMI, WC, and WHR and these results are presented in Supplementary Tables 8–10. Smoking statuses included never smokers, former smokers, and current smokers. The risk of developing lung cancer during the follow-up period increased among former and current smokers (versus never smokers) but among current smokers, HRs were highest among the smallest category of body shape and size (i.e. normal BMI, below guidelines for WC, and the lowest quartile of WHR).

## Discussion

In this prospective cohort of over 25,000 participants, males and females classified as obese (BMI ≥ 30 kg/m^2^) had a meaningfully increased risk of all-cancer, respectively, compared to their normal weight counterparts. Similar increases in risk of all-cancer were observed for those participants with WC above sex-specific guidelines. While our results indicate that both high BMI and high WC increase the risk of all-cancer, mutual adjustment for WC attenuated the association between BMI and all-cancer risk, especially among females. This finding suggests that central adiposity, as measured by WC, is a stronger predictor of all-cancer risk than BMI. Having a WHR in the highest versus the lowest quartile also increased the risk of all-cancers among both males and females.

In this cohort, having a greater BMI or WC above guidelines increased the risks of colon cancer, non-Hodgkin lymphoma and hematological cancers among males. A positive dose–response effect for increasing quartiles of WHR and the risk of prostate cancer was detected in males in the highest quartile, who had a 42% increased risk. Among females, we found strong evidence of an increased risk of endometrial cancer with higher values for all three anthropometric measures (BMI, WC, and WHR). While no statistically significant associations between the three measures of body shape and size and the risk of postmenopausal breast cancer were detected, the direction of risk was reversed by menopausal status; postmenopausal women with a BMI ≥ 30 kg/m^2^ had an increased risk of breast cancer and premenopausal women with a BMI ≥ 30 kg/m^2^ had a decreased risk of breast cancer. Interestingly, a statistically significant inverse association between higher BMI and the risk of lung cancer was observed among females, which was in the same direction but not statistically significant among males.

The most common universal hypothesis explaining the molecular mechanism of obesity that promotes cancer is insulin resistance^22^. Elevated concentrations of insulin are believed to activate the insulin-like growth factor pathway which, in turn, stimulates the growth and proliferation of cells and inhibits apoptosis, subsequently leading to tumor development^23^. Sex hormones, particularly estrogens, have been implicated in the relationship between body size and shape, and some site-specific cancers. For instance, following menopause estrogens are mainly produced in adipose tissue and rising estrogens in the blood are believed to encourage the development of breast and endometrial cancers via multiple pathways^22^. Mechanisms linking body size and shape to cancer pathogenesis continue to emerge and evolve including the concepts of obesity-induced hypoxia, shared genetic susceptibility, and the migration of adipose stromal cells^24^.

Our findings related to BMI and overall cancer risk within the ATP cohort are generally in agreement with the existing literature base. Systematic reviews and meta-analyses have also detected a stronger association between increasing BMI and the risk of colon cancer among males than females, although these sex differences are not fully understood^25–27^. Previous studies report that increasing BMI is protective factor against the incidence of lung cancer in both males and females^26–29^ and we observed a statistically significant inverse trend between increasing BMI and lung cancer for females, which was in the same direction for males. This inverse association appears to be strongest among current smokers; however, we did not present such stratified analyses by sex because of the low statistical power. Existing literature indicates a mild positive association between increasing BMI and postmenopausal breast cancer^26,27^ and while our findings were not statistically significant, they were in the positive direction for women with a BMI ≥ 30 kg/m^2^. Consistent with our findings, strong positive associations between increasing BMI and endometrial cancer risk have been reported in numerous studies^18,26,27,30^.

Given that BMI has been criticized for not providing any indication of the fat distribution^31^, it is valuable to consider additional indicators of body shape and size including WC and WHR. Interestingly, we found differences in the direction of risk for premenopausal breast when considering BMI versus WHR; higher BMI was associated with a reduced risk of breast cancer whereas those in the second and third quartiles of WHR had an increased risk of breast cancer and those in the fourth quartile of WHR had a decreased risk of breast cancer, though these estimates were not statistically significant. While some studies have associated increased central obesity and WHR with adverse risk among premenopausal women^32–34^, this association has not been consistently observed^35^, and a meta-analysis of central obesity found no overall effect in premenopausal women^36^. Mutations in the breast cancer genes (BRCA) 1 and 2 increase the risk of developing young-onset breast cancer;^37,38^ however, only a small number of breast cancers diagnosed among young women (~5–10%) are attributable to these mutations^38^, suggesting that other unknown genetic, lifestyle, or environmental factors are involved.

Several limitations to the data presented must be acknowledged. First, participants who were enrolled in our sample were not diverse in terms of some sociodemographic variables^39^ and thus, are likely not representative of the entire Alberta population. For example, the majority of our sample was female (62%) and Caucasian (91%). Second, the ATP cohort has a higher proportion of participants who have a BMI ≥ 25 kg/m^2^ compared to participants in the same age range (35–69 years) from Alberta who participated in Cycle 1.1 of the Canadian Community Health Survey (CCHS) collected in 2005, available from Statistics Canada; 75.3% of males and 59.2% of females from the present analyses were classified as overweight or obese versus 65.4% of males and 48.1% of females in the CCHS subsample^40^. Accordingly, the generalizability of our findings is limited. Third, although we included cancer sites with ≥100 incident cases, there were small cell counts in some cases after body shape and size variables were divided into relevant categories. This sample size resulted in some less precise site-specific estimates that should be interpreted with caution (e.g. leukemia and Non-Hodgkin lymphoma). Fourth, the use of self-reported measures of height, weight and waist and hip circumferences that were used to derive our exposure variables may have been influenced by a social desirability bias, which may have introduced non-differential misclassification bias. Studies to validate these particular measures should be considered going forward. Furthermore, future studies examining the relationship between body shape and size, and cancer risk should consider using more objective measures of adiposity such as dual-energy X-ray absorptiometry, which are not prone to information biases. Finally, we assumed that our exposure variables remained constant during the follow-up period; however, given that several studies have found that BMI tends to increase into early old age^41–43^, our estimates are likely underestimations of the true HRs.

Strengths of this paper include: (i) the detailed instructions in the HLQ and measuring tape provided to participants in order to collect their measurements, which likely improved the accuracy of self-reporting, (ii) the availability of data on all-cancer as well as eight site-specific cancers in the same cohort, and (iii) the sensitivity analyses performed that excluded cancers occurring <2 years after baseline data collection were removed to decrease the possibility of reverse causation, and (iv) the ability to establish temporality through the use of a large prospective cohort study.

These analyses suggest that central adiposity is a strong predictor of all-cancer risk, especially among females and highlight the importance of weight distribution. Public health interventions that promote weight management via multifaceted approaches can contribute to cancer primary prevention. Further, secondary cancer prevention measures may be improved by identifying targeting screening programs to individuals who are at higher risk for developing cancer due to the distribution of their body weight. We recommend that future research studies investigate changes in body shape and size over time related to cancer risk and use more accurate measures of body size and weight distribution.

## Methods

### Data source

We analyzed data from ATP, a prospective cohort study of adults in the province of Alberta, Canada. The ATP, which started in 2000, aims to investigate factors that influence cancer and chronic disease risk. A full description of the study design and recruitment for ATP has been previously published^39^. In brief, participants were recruited through eight waves of telephone-based random digit dialing using regional health authority boundaries within the province of Alberta. Participants were eligible if they were between the ages of 35 and 69 years, had no previous history of cancer (other than non-melanoma skin cancer), planned to reside in Alberta for at least one year, were not pregnant at the time of enrollment, and were able to complete questionnaires in English.

**Fig. 3:**
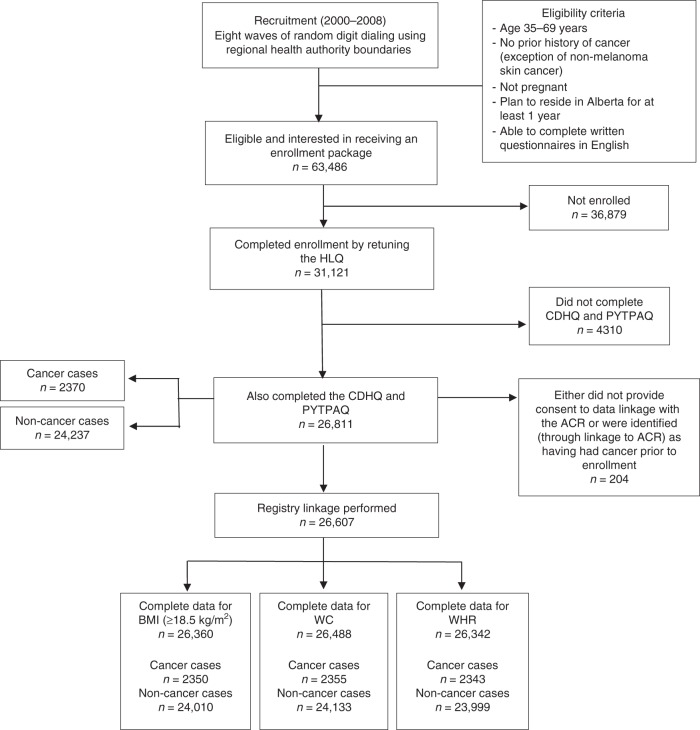
Recruitment, enrollment, and analytic sample selection flow diagram for Alberta’s Tomorrow Project, resulting in an analytic sample of 26,607 participants

As presented in Fig. 3, a total of 63,486 participants (42.1% male, 57.9% female) were eligible and expressed interest in receiving an enrollment package. Of these participants, 49% (*n* = 31,121) enrolled in ATP and completed the Health and Lifestyle Questionnaire (HLQ). The HLQ included domains related to socio-demographics, personal and family health history, lifestyle, and anthropometric measurements. Twelve weeks after completing the HLQ, participants were mailed the Canadian Diet History Questionnaire (CDHQ) and the Past Year Total Physical Activity Questionnaire (PYTPAQ). The present study focused on a subset of the cohort who completed the HLQ, CDHQ, and PYTPAQ and consented to data linkage with administrative databases, including the provincial cancer registry (Fig. 3).

Ethical approval for recruitment and data collection was approved by the former Alberta Cancer Board Research Ethics Committee and the University of Calgary Conjoint Health Research Ethics Board. Ethical approval for the current study was obtained from the Health Research Ethics Board of Alberta—Cancer Committee. Written informed consent was obtained from all study participants.

### Body size and shape assessment

The HLQ included a section that instructed participants on how to take accurate baseline measurements of their height, weight, abdominal circumference, and buttocks circumference. Participants were provided with a tape measure (divided in 1/8ʺ sections) and were instructed to use a scale, set to zero, to measure their current weight. All measurements were to be made in a single session at least 2 h after eating a meal, and preferably with the help of another adult. For height, participants were instructed to remove their shoes, stand straight with their back and heels against the wall and record their height in feet and inches. For weight, participants were instructed to remove their shoes, wear light clothing and record their weight in pounds. Instructions for abdominal circumference stated, “Measure one inch above your navel or belly button, even if this is not your usual waistline”. For buttocks circumference, participants were instructed to “Slide the tape measure up and down until you find the largest spot between your waist and thighs”. Diagrams were included to aid in identifying the correct measurement location for the abdomen and buttocks. Participants were instructed to take at least two measurements for each anthropometric measure. Prior to deriving single variables for height, weight, BMI, abdominal and buttocks circumference, research staff at ATP applied range checks to body measurements and contacted participants in the case of missing, contradictory, or extreme measurements.

We considered BMI (kg/m^2^) as a continuous variable and as a categorical variable classified as normal weight (BMI 18.5 to < 25 kg/m^2^), overweight (BMI 25 to <30 kg/m^2^), and obese (BMI ≥ 30 kg/m^2^). Given the U-shaped relationship between BMI and all-cause mortality that has been observed in several populations^44,45^, we decided to exclude individuals (*n* = 181) classified as underweight (BMI < 18.5 kg/m^2^) rather than combining them with those classified as normal weight. WC was also considered as a continuous and categorical variable using the World Health Organization (WHO) cut-off points for a substantially increased risk of metabolic complications: ≥102 cm for males and ≥88 cm for females^46^. We planned to examine categories of WHR based on the WHO cut-offs points for a substantially increased risk of metabolic complications (≥0.90 cm for males ≥0.85 cm for females)^46^; however, since 84% of males in our sample had a WHR above the recommended WHO guideline, we examined quartiles of WHR instead.

### Cancer registry linkage

Participants’ Personal Health Numbers were linked with the Alberta Cancer Registry (ACR) to identify incident, primary cancers (with the exception of non-melanoma skin cancers) up to June 2017. The coding of new cancer cases by site is based on the International Classification of Diseases for Oncology, Third Edition^47^. The ACR received a gold level certification status under the North American Association of Central Registries from 2002 to 2013, indicating that cancer case ascertainment was ≥95%^48^.

We considered all incident, primary cancer cases as well as eight site-specific cancers where ≥100 incident cases occurred in the cohort: breast, colon (includes cancers of the colon and recto-sigmoid junction), prostate, lung, endometrial, non-Hodgkin lymphoma, leukemia, and hematological cancers (includes Hodgkin lymphoma, non-Hodgkin lymphoma, leukemia, multiple myeloma and plasmacytoma, and other hematopoietic and reticuloendothelial cancers).

### Covariates

We adjusted for multiple covariates based on known associations with both body shape and size, and cancer. All adjusted models included the following variables: age, sex, self-reported ethnicity (white/other), marital status (married or living with someone/divorced, separated, or widowed/single), highest education level (high school or less/some post-high school education/post-high school certificate or degree), total household income ($0–$49,999/$50,000–$99,999/≥$100,000), geographical area of residence (urban/rural), smoking status (never/former/current), mean energy intake (kilocalories per day), mean alcohol intake (grams of ethanol per day), total weekly metabolic output from all activities (metabolic equivalents of task [MET]—hours per week), ever being diagnosed with diabetes by a physician (yes/no), and family history of cancer (yes/no). Other covariates adjusted for in site-specific models include: menopausal status (pre-menopause/post-menopause) and oral contraceptive use (0–5 years/>5 years) for breast and endometrial cancers; pack-years of cigarette smoking for lung cancer; and mean fiber intake (grams per day) for colon cancer. We included a dichotomous (yes/no) variable for indicating ever having cancer screening tests in adjusted models for breast cancer (physical breast exam and/or mammogram), colon cancer (blood stool test and/or sigmoidoscopy or colonoscopy), and prostate cancer (digital rectal exam and/or prostate-specific antigen test). Lastly, for breast cancer, we included a dichotomous (yes/no) variable for ever having used female hormones (tablets, pills or creams) for menopause.

### Statistical analyses

Participant follow-up time was calculated from entry into the study (based on exact age at the time of HLQ completion) to date of cancer diagnosis for cases (based on exact age at diagnosis), or to the end of follow-up for non-cancer cases (based on their exact age at the time of data linkage with ACR in June 2017). Descriptive statistics were used to characterize the cohort by sex and cancer incidence status. We used Cox proportional hazards models to compute age-adjusted and multivariate-adjusted hazards ratios (HR) and 95% confidence intervals (CIs) of all-cancer and site-specific cancer incidence for the three measures of body shape and size (BMI, WC, and WHR) for males and females, separately. Unless otherwise noted, the HRs we reported are from the multivariate-adjusted models. We conducted sensitivity analyses, where incident cancers occurring less than two years after baseline data collection were excluded, in order to minimize the potential of reverse causation (i.e. a participant’s body shape and size being influenced by an existing, yet undiagnosed cancer). Tests of linear trend were performed by scoring the categories of the various measures of body shape and size and imputing the score as a continuous term in the Cox proportional hazard model and reporting the associated *P* values of the Wald *Z*-tests. For all analyses, a two-tailed *P*-value of <0.05 was considered statistically significant and Stata software (version 14.2) was used^49^.

## Supplementary information

Supplementary Tables

## Data Availability

All associated code, protocols, additional results and/or aggregated data from this study are available from the corresponding author upon reasonable request. Additional access to individual-level data is available in accordance with the Health Information Act of Alberta and the data access guidelines of Alberta’s Tomorrow Project at https://myatp.ca/. The data from this study included all participants from Phase I of cohort data collection accessed under accession code Brenner-2016–04.
