# Supplementary material for: Central body fatness is a stronger predictor of cancer risk than overall body size
Source: Nat Commun. 2019 Jan 22;10:383. doi: 10.1038/s41467-018-08159-w (PMC6342989; doi:10.1038/s41467-018-08159-w)
Supplement: Supplementary file 1 — Supplementary Tables [file 41467_2018_8159_MOESM1_ESM.pdf]

Supplementary Information

Central body fatness is a stronger predictor of cancer risk than overall body size

Barberio et al.

Supplementary Tables:

Supplementary Table 1. Results from analyses of BMI categories[1] and cancer incidence for cancers occurring greater than two years after baseline data collection (n=26 078)

	MALES (n=9 861)		FEMALES (n=16 217)	
	Cases	Latency Multivariate-adjusted[2]	Cases	Latency Multivariate-adjusted[2]
All-cancers				
Normal	157	1.0 (Ref)	390	1.0 (Ref)
Overweight	404	1.10 (0.91, 1.32)	379	1.0 (0.86, 1.15)
Obese	301	1.43** (1.17, 1.74)	373	1.26** (1.08, 1.46)
P for trend		<0.001		<0.01
Prostate cancer				
Normal	63	1.0 (Ref)	--	--
Overweight	174	1.15 (0.86, 1.54)	--	--
Obese	101	1.20 (0.87, 1.65)	--	--
P for trend		0.29		--
Breast cancer – Premenopausal				
Normal	--	--	87	1.0 (Ref)
Overweight	--	--	47	0.78 (0.54, 1.11)
Obese	--	--	33	0.76 (0.50, 1.15)
P for trend		--		0.14
Breast cancer – Postmenopausal				
Normal	--	--	77	1.0 (Ref)
Overweight	--	--	78	0.90 (0.65, 1.23)
Obese	--	--	85	1.21 (0.88, 1.67)
P for trend		--		0.25
Endometrial cancer				
Normal	--	--	16	1.0 (Ref)
Overweight	--	--	24	1.73† (0.92, 3.28)
Obese	--	--	57	4.98** (2.81, 8.85)
P for trend		--		<0.001
Colon cancer				
Normal	7	1.0 (Ref)	21	1.0 (Ref)
Overweight	32	1.82 (0.80, 4.14)	42	1.99** (1.17, 3.38)
Obese	34	3.20** (1.40, 7.32)	27	1.66† (0.92, 2.99)
P for trend		<0.001		0.09
Lung cancer				
Normal	16	1.0 (Ref)	47	1.0 (Ref)
Overweight	25	0.70 (0.37, 1.33)	42	0.79 (0.52, 1.21)
Obese	19	0.83 (0.41, 1.65)	26	0.61* (0.37, 1.00)
P for trend		0.65		0.05
Leukemia				
Normal	11	1.0 (Ref)	13	1.0 (Ref)
Overweight	21	0.89 (0.42, 1.86)	14	1.01 (0.47, 2.17)
Obese	15	1.16 (0.52, 2.57)	8	0.69 (0.28, 1.73)
P for trend		0.68		0.46
Non-Hodgkin lymphoma				
Normal	7	1.0 (Ref)	10	1.0 (Ref)
Overweight	17	1.11 (0.46, 2.71)	15	1.46 (0.65, 3.28)
Obese	24	2.60* (1.10, 6.15)	15	1.82 (0.80, 4.15)
P for trend		0.01		0.15
Hematological cancers				
Normal	19	1.0 (Ref)	24	1.0 (Ref)
Overweight	39	0.94 (0.54, 1.64)	30	1.22 (0.71, 2.10)
Obese	39	1.64† (0.93, 2.88)	24	1.23 (0.68, 2.20)
P for trend		0.04		0.49

Note: Hazard ratios (HRs) and 95% confidence intervals (CIs) from Cox regression models are presented
[1]Normal = ≥18.5 to < 25 kg/m^2; Overweight = ≥ 25 to <30 kg/m^2; Obese = ≥ 30 kg/m^2
Those with a BMI <18.5 kg/m^2 were excluded from these analyses
[2]Adjusted for: age (continuous), ethnicity (white/other), marital status (married or living with someone/divorced, separated, or widowed/single, never married), highest level of education (high school or less/some post-high school education/post-high school certificate or degree), total household income ($0 to $49,999/$50,000 to $99,999/≥ $100,000), geographical area of residence (urban/rural), smoking status (current/former/never), alcohol consumption (grams of ethanol per day), energy intake (kilocalories per day), total physical activity (MET-hours per week), history of diabetes (yes/no), family history of cancer (yes/no), pack-years of cigarettes (lung cancer only), fibre intake (grams per day) (colon cancer only), menopausal status (pre-menopause/post-menopause) (endometrial cancers), years of birth control use (0 to 5 years/>5 years), (breast and endometrial cancers), history of breast cancer screening (yes/no) (breast cancer only), history of colon cancer screening (yes/no) (colon cancer only), and history of prostate cancer screening (yes/no) (prostate cancer only)
**p<0.01, *p<0.05, †p<0.1

Supplementary Table 2. Results from analyses of BMI categories[1] and cancer incidence among ATP participants with BMI ≥18.5 kg/m^2 adjusting for all covariates and dichotomous WC (n=25 499)

	MALES (n=9 749)				FEMALES (n=15 750)			
	Cases	Multivariate-adjusted[2]	Cases	Multivariate-adjusted[3]	Cases	Multivariate-adjusted[2]	Cases	Multivariate-adjusted[3]
All-cancer								
Normal	185	1.0 (Ref)	185	1.0 (Ref)	447	1.0 (Ref)	447	1.0 (Ref)
Overweight	471	1.08 (0.91, 1.29)	471	1.04 (0.87, 1.25)	418	0.95 (0.83, 1.09)	416	0.91 (0.78, 1.06)
Obese	331	1.33** (1.10, 1.60)	328	1.20 (0.94, 1.52)	420	1.22** (1.06, 1.40)	416	1.11 (0.90, 1.35)
P for trend		<0.001		0.12		<0.01		0.30
Prostate cancer								
Normal	72	1.0 (Ref)	72	1.0 (Ref)	--	--	--	--
Overweight	205	1.17 (0.89, 1.53)	205	1.12 (0.84, 1.49)	--	--	--	--
Obese	115	1.17 (0.87, 1.58)	114	1.03 (0.71, 1.51)	--	--	--	--
P for trend		0.34		0.91		--		--
Breast cancer – Premenopausal								
Normal	--	--	--	--	98	1.0 (Ref)	98	1.0 (Ref)
Overweight	--	--	--	--	50	0.73† (0.52, 1.04)	50	0.65* (0.43, 0.97)
Obese	--	--	--	--	34	0.69† (0.46, 1.04)	34	0.53* (0.30, 0.95)
P for trend		--		--		0.04		0.02
Breast cancer - Postmenopausal								
Normal	--	--	--	--	85	1.0 (Ref)	85	1.0 (Ref)
Overweight	--	--	--	--	89	0.92 (0.69, 1.25)	89	0.90 (0.63, 1.27)
Obese	--	--	--	--	99	1.26 (0.93, 1.71)	99	1.20 (0.77, 1.85)
P for trend		--		--		0.13		0.36
Endometrial cancer								
Normal	--	--	--	--	20	1.0 (Ref)	20	1.0 (Ref)
Overweight	--	--	--	--	26	1.48 (0.82, 2.67)	26	1.23 (0.62, 2.42)
Obese	--	--	--	--	65	4.52** (2.69, 7.61)	64	3.12** (1.43, 6.81)
P for trend		--		--		<0.001		<0.001
Colon cancer								
Normal	9	1.0 (Ref)	9	1.0 (Ref)	26	1.0 (Ref)	26	1.0 (Ref)
Overweight	37	1.66 (0.79, 3.45)	37	1.56 (0.72, 3.38)	48	1.80* (1.11, 2.92)	48	1.80* (1.05, 3.08)
Obese	36	2.71** (1.28, 5.70)	35	2.28† (0.92, 5.67)	32	1.49 (0.87, 2.56)	32	1.48 (0.73, 3.00)
P for trend		<0.001		0.07		0.14		0.33
Lung cancer								
Normal	18	1.0 (Ref)	18	1.0 (Ref)	51	1.0 (Ref)	51	1.0 (Ref)
Overweight	32	0.78 (0.43, 1.41)	32	0.52† (0.25, 1.07)	46	0.80 (0.53, 1.19)	46	0.82 (0.51, 1.31)
Obese	19	0.71 (0.37, 1.39)	19	0.36* (0.14, 0.90)	28	0.59* (0.36, 0.95)	28	0.61 (0.32, 1.16)
P for trend		0.33		0.03		0.03		0.14
Leukemia								
Normal	12	1.0 (Ref)	12	1.0 (Ref)	17	1.0 (Ref)	17	1.0 (Ref)
Overweight	23	0.88 (0.43, 1.78)	23	0.82 (0.38, 1.75)	14	0.76 (0.37, 1.55)	14	0.57 (0.24, 1.36)
Obese	17	1.15 (0.54, 2.46)	17	0.96 (0.35, 2.65)	11	0.68 (0.31, 1.52)	11	0.42 (0.14, 1.27)
P for trend		0.67		0.94		0.33		0.13
Non-Hodgkin lymphoma								
Normal	8	1.0 (Ref)	8	1.0 (Ref)	13	1.0 (Ref)	13	1.0 (Ref)
Overweight	19	1.08 (0.47, 2.49)	19	0.94 (0.39, 2.30)	16	1.20 (0.57, 2.50)	16	1.41 (0.63, 3.16)
Obese	26	2.47* (1.10, 5.57)	26	1.77 (0.60, 5.16)	16	1.46 (0.69, 3.11)	16	2.07 (0.70, 6.11)
P for trend		0.01		0.18		0.32		0.19
Hematological cancers								
Normal	21	1.0 (Ref)	21	1.0 (Ref)	31	1.0 (Ref)	31	1.0 (Ref)
Overweight	43	0.93 (0.55, 1.58)	43	0.85 (0.49, 1.50)	31	0.96 (0.58, 1.59)	31	0.91 (0.51, 1.63)
Obese	43	1.61† (0.94, 2.75)	43	1.28 (0.62, 2.62)	28	1.05 (0.62, 1.79)	28	0.94 (0.44, 2.01)
P for trend		0.04		0.41		0.86		0.88

Note: Hazard ratios (HRs) and 95% confidence intervals (CIs) from Cox regression models are presented

[1]Normal = ≥18.5 to < 25 kg/m^2; Overweight = ≥ 25 to <30 kg/m^2; Obese = ≥ 30 kg/m^2
Those with a BMI <18.5 kg/m^2 were excluded from these analyses
[2]Adjusted for: age (continuous), ethnicity (white/other), marital status (married or living with someone/divorced, separated, or widowed/single, never married), highest level of education (high school or less/some post-high school education/post-high school certificate or degree), total household income ($0 to $49,999/$50,000 to $99,999/≥ $100,000), geographical area of residence (urban/rural), smoking status (current/former/never), alcohol consumption (grams of ethanol per day), energy intake (kilocalories per day), total physical activity (MET-hours per week), history of diabetes (yes/no), family history of cancer (yes/no), pack-years of cigarettes (lung cancer only), fibre intake (grams per day) (colon cancer only), menopausal status (pre-menopause/post-menopause) (endometrial cancers), years of birth control use (0 to 5 years/>5 years), (breast and endometrial cancers), history of breast cancer screening (yes/no) (breast cancer only), history of colon cancer screening (yes/no) (colon cancer only), and history of prostate cancer screening (yes/no) (prostate cancer only)
[3]Adjusted for: all covariates listed above and waist circumference (below/above guidelines)
**p<0.01, *p<0.05, †p<0.1

Supplementary Table 3. Results from analyses of waist circumference[1] and cancer incidence for cancers occurring greater than two years after baseline data collection (n=26,205)

	MALES (n=9 864)		FEMALES (n=16 341)	
	Cases	Latency multivariate-adjusted[2]	Cases	Latency multivariate-adjusted[2]
All-cancers				
Below	417	1.0 (Ref)	545	1.0 (Ref)
Above	447	1.28** (1.12, 1.47)	600	1.20** (1.06, 1.35)
Prostate cancer				
Below	176	1.0 (Ref)	--	--
Above	163	1.14 (0.91, 1.42)	--	--
Breast cancer – Premenopausal				
Below	--	--	109	1.0 (Ref)
Above	--	--	61	0.95 (0.69, 1.31)
Breast cancer – Postmenopausal				
Below	--	--	110	1.0 (Ref)
Above	--	--	131	1.11 (0.85, 1.44)
Endometrial				
Below	--	--	26	1.0 (Ref)
Above	--	--	71	3.21** (2.01, 5.12)
Colon				
Below	25	1.0 (Ref)	40	1.0 (Ref)
Above	47	2.01** (1.22, 3.32)	51	1.36 (0.88, 2.09)
Lung				
Below	26	1.0 (Ref)	60	1.0 (Ref)
Above	36	1.23 (0.73, 2.09)	57	0.78 (0.53, 1.14)
Leukemia				
Below	24	1.0 (Ref)	16	1.0 (Ref)
Above	23	1.35 (0.75, 2.42)	19	1.24 (0.62, 2.47)
Non-Hodgkin Lymphoma				
Below	18	1.0 (Ref)	18	1.0 (Ref)
Above	30	2.10* (1.15, 3.83)	22	1.21 (0.64, 2.31)
Hematological				
Below	44	1.0 (Ref)	35	1.0 (Ref)
Above	53	1.60* (1.06, 2.42)	43	1.29 (0.82, 2.06)

Note: Hazard ratios (HRs) and 95% confidence intervals (CIs) from Cox regression models are presented
[1]waist circumference below guidelines: <102 cm for men, < 88 cm for women
[1]waist circumference above guidelines: ≥102 cm for men, ≥88 cm for women
[2]Adjusted for: age (continuous), ethnicity (white/other), marital status (married or living with someone/divorced, separated, or widowed/single, never married), highest level of education (high school or less/some post-high school education/post-high school certificate or degree), total household income ($0 to $49,999/$50,000 to $99,999/≥ $100,000), geographical area of residence (urban/rural), smoking status (current/former/never), alcohol consumption (grams of ethanol per day), energy intake (kilocalories per day), total physical activity (MET-hours per week), history of diabetes (yes/no), family history of cancer (yes/no), pack-years of cigarettes (lung cancer only), fibre intake (grams per day) (colon cancer only), menopausal status (pre-menopause/post-menopause) (endometrial cancer), years of birth control use (0 to 5 years/>5 years), (breast and endometrial cancers), history of breast cancer screening (yes/no) (breast cancer only), history of colon cancer screening (yes/no) (colon cancer only), and history of prostate cancer screening (yes/no) (prostate cancer only)
**p<0.01, *p<0.05, †p<0.1

Supplementary Table 4. Results from analyses of waist circumference[1] and cancer incidence adjusting for all covariates and dichotomous BMI (n=25 499)

	MALES (n=9 749)				FEMALES (n=15 750)			
	Cases	Multivariate-adjusted[2]	Cases	Multivariate-adjusted[3]	Cases	Multivariate-adjusted[2]	Cases	Multivariate-adjusted[3]
All-cancer								
Below	488	1.0 (Ref)	485	1.0 (Ref)	620	1.0 (Ref)	609	1.0 (Ref)
Above	499	1.22** (1.07, 1.38)	499	1.20* (1.04, 1.39)	671	1.17** (1.04, 1.31)	670	1.21** (1.04, 1.40)
Prostate cancer								
Below	202	1.0 (Ref)	201	1.0 (Ref)	--	--	--	--
Above	190	1.14 (0.93, 1.39)	190	1.10 (0.88, 1.38)	--	--	--	--
Breast cancer – Premenopausal								
Below	--	--	--	--	121	1.0 (Ref)	118	1.0 (Ref)
Above	--	--	--	--	64	0.90 (0.66, 1.23)	64	1.24 (0.80, 1.90)
Breast cancer – Postmenopausal								
Below	--	--	--	--	120	1.0 (Ref)	119	1.0 (Ref)
Above	--	--	--	--	154	1.18 (0.93, 1.52)	154	1.22 (0.89, 1.67)
Endometrial cancer								
Below	--	--	--	--	30	1.0 (Ref)	30	1.0 (Ref)
Above	--	--	--	--	81	3.14** (2.04, 4.85)	80	2.41** (1.38, 4.22)
Colon cancer								
Below	32	1.0 (Ref)	32	1.0 (Ref)	48	1.0 (Ref)	47	1.0 (Ref)
Above	49	1.67* (1.06, 2.65)	49	1.44 (0.88, 2.38)	59	1.25 (0.84, 1.86)	59	0.93 (0.58, 1.50)
Lung cancer								
Below	30	1.0 (Ref)	28	1.0 (Ref)	67	1.0 (Ref)	63	1.0 (Ref)
Above	41	1.20 (0.73, 1.96)	41	1.85† (0.95, 3.58)	62	0.74 (0.51, 1.06)	62	0.87 (0.54, 1.40)
Leukemia								
Below	27	1.0 (Ref)	27	1.0 (Ref)	20	1.0 (Ref)	20	1.0 (Ref)
Above	25	1.25 (0.71, 2.18)	25	1.34 (0.71, 2.54)	22	1.08 (0.57, 2.03)	22	1.58 (0.66, 3.78)
Non-Hodgkin lymphoma								
Below	20	1.0 (Ref)	20	1.0 (Ref)	22	1.0 (Ref)	22	1.0 (Ref)
Above	33	2.11** (1.19, 3.75)	33	2.10* (1.09, 4.04)	23	1.05 (0.57, 1.92)	23	0.83 (0.40, 1.73)
Hematological cancers								
Below	49	1.0 (Ref)	49	1.0 (Ref)	43	1.0 (Ref)	43	1.0 (Ref)
Above	58	1.56* (1.05, 2.30)	58	1.63* (1.04, 2.55)	47	1.12 (0.73, 1.71)	47	1.16 (0.67, 2.02)

Note: Hazard ratios (HRs) and 95% confidence intervals (CIs) from Cox regression models are presented
[1]waist circumference below guidelines: <102 cm for men, < 88 cm for women
[1]waist circumference above guidelines: ≥102 cm for men, ≥88 cm for women
[2]Adjusted for: age (continuous), ethnicity (white/other), marital status (married or living with someone/divorced, separated, or widowed/single, never married), highest level of education (high school or less/some post-high school education/post-high school certificate or degree), total household income ($0 to $49,999/$50,000 to $99,999/≥ $100,000), geographical area of residence (urban/rural), smoking status (current/former/never), alcohol consumption (grams of ethanol per day), energy intake (kilocalories per day), total physical activity (MET-hours per week), history of diabetes (yes/no), family history of cancer (yes/no), pack-years of cigarettes (lung cancer only), fibre intake (grams per day) (colon cancer only), menopausal status (pre-menopause/post-menopause) (endometrial cancer), years of birth control use (0 to 5 years/>5 years), (breast and endometrial cancers), history of breast cancer screening (yes/no) (breast cancer only), history of colon cancer screening (yes/no) (colon cancer only), and history of prostate cancer screening (yes/no) (prostate cancer only)
[3]Adjusted for: all variables in multivariate-analysis[2] as well as BMI (≥18.5 kg/m^2 and < 25 kg/m^2/ = ≥ 25 kg/m^2 and < 30 kg/m^2/ = ≥ 30 kg/m^2).
**p<0.01, *p<0.05, †p<0.1

Supplementary Table 5. Results from analyses of waist circumference[1] in quartiles and cancer incidence (n=26 488)

	MALES (n=9 990)						FEMALES (n=16 498)					
	Cases	Age-adjusted	Cases	Multivariate-adjusted[2]	Cases	Multivariate-adjusted[3]	Cases	Age-adjusted	Cases	Multivariate-adjusted[2]	Cases	Multivariate-adjusted[3]
All-cancer												
1st Quartile	30	1.0 (Ref)	28	1.0 (Ref)	26	1.0 (Ref)	425	1.0 (Ref)	408	1.0 (Ref)	398	1.0 (Ref)
2nd Quartile	155	0.83 (0.56, 1.23)	152	0.86 (0.58, 1.29)	152	0.90 (0.59, 1.37)	361	1.08 (0.94, 1.24)	346	1.07 (0.93, 1.24)	345	1.11 (0.94, 1.32)
3rd Quartile	342	0.92 (0.64, 1.34)	336	0.95 (0.64, 1.40)	335	0.97 (0.63, 1.50)	276	1.12 (0.96, 1.30)	262	1.09 (0.93, 1.27)	262	1.09 (0.88, 1.36)
4th Quartile	476	1.11 (0.77, 1.61)	465	1.12 (0.76, 1.66)	465	1.08 (0.68, 1.50)	282	1.37** (1.17, 1.59)	273	1.34** (1.14, 1.58)	272	1.26† (0.97, 1.64)
P for trend		<0.01		<0.01		0.20		<0.01		<0.01		0.11
Prostate cancer												
1st Quartile	12	1.0 (Ref)	11	1.0 (Ref)	10	1.0 (Ref)	--	--	--	--	--	--
2nd Quartile	66	0.86 (0.46, 1.59)	66	0.91 (0.48, 1.73)	66	0.94 (0.48, 1.84)	--	--	--	--	--	--
3rd Quartile	142	0.92 (0.51, 1.67)	139	0.93 (0.50, 1.72)	139	0.89 (0.44, 1.78)	--	--	--	--	--	--
4th Quartile	178	0.99 (0.55, 1.78)	174	1.02 (0.55, 1.90)	174	1.00 (0.48, 2.07)	--	--	--	--	--	--
P for trend		0.42		0.44		0.72	--	--	--	--	--	--
Breast cancer – Premenopausal												
1st Quartile	--	--	--	--	--	--	94	1.0 (Ref)	91	1.0 (Ref)	88	1.0 (Ref)
2nd Quartile	--	--	--	--	--	--	47	0.89 (0.63, 1.27)	45	0.87 (0.61, 1.25)	45	1.17 (0.76, 1.79)
3rd Quartile	--	--	--	--	--	--	30	0.97 (0.64, 1.46)	27	0.89 (0.58, 1.37)	27	1.54 (0.84, 2.80)
4th Quartile	--	--	--	--	--	--	23	0.95 (0.60, 1.50)	22	0.89 (0.55, 1.44)	22	1.82 (0.82, 4.01)
P for trend	--	--	--	--	--	--		0.78		0.51		0.11
Breast cancer – Postmenopausal												
1st Quartile	--	--	--	--	--	--	79	1.0 (Ref)	74	1.0 (Ref)	73	1.0 (Ref)
2nd Quartile	--	--	--	--	--	--	83	1.11 (0.82, 1.52)	82	1.19 (0.87, 1.63)	82	1.18 (0.82, 1.72)
3rd Quartile	--	--	--	--	--	--	63	1.06 (0.76, 1.48)	60	1.11 (0.78, 1.56)	60	0.92 (0.57, 1.50)
4th Quartile	--	--	--	--	--	--	61	1.20 (0.86, 1.67)	58	1.24 (0.87, 1.78)	58	0.88 (0.50, 1.53)
P for trend	--	--	--	--	--	--		0.37		0.31		0.54
Endometrial cancer												
1st Quartile	--	--	--	--	--	--	23	1.0 (Ref)	23	1.0 (Ref)	23	1.0 (Ref)
2nd Quartile	--	--	--	--	--	--	19	1.12 (0.61, 2.07)	15	0.94 (0.49, 1.81)	15	0.63 (0.29, 1.38)
3rd Quartile	--	--	--	--	--	--	32	2.66** (1.55, 4.57)	27	2.34** (1.32, 4.13)	27	1.06 (0.45, 2.48)
4th Quartile	--	--	--	--	--	--	47	4.68** (2.82, 7.76)	45	4.48** (2.63, 7.63)	44	1.53 (0.61, 3.85)
P for trend	--	--	--	--	--	--		<0.01		<0.01		0.11
Colon cancer												
1st Quartile	1	1.0 (Ref)	1	1.0 (Ref)	1	1.0 (Ref)	29	1.0 (Ref)	28	1.0 (Ref)	28	1.0 (Ref)
2nd Quartile	5	0.80 (0.09, 6.86)	5	0.74 (0.09, 6.38)	5	0.70 (0.08, 6.06)	29	1.23 (0.73, 2.07)	29	1.26 (0.75, 2.13)	28	0.89 (0.48, 1.66)
3rd Quartile	29	2.33 (0.32, 17.20)	29	2.03 (0.27, 15.07)	29	1.79 (0.22, 14.62)	26	1.46 (0.86, 2.50)	26	1.47 (0.85, 2.54)	26	1.13 (0.55, 2.32)
4th Quartile	45	3.13 (0.43, 22.82)	44	2.47 (0.33, 18.23)	44	1.77 (0.20, 15.46)	24	1.63† (0.94, 2.81)	24	1.61 (0.90, 2.86)	24	1.49 (0.63, 3.55)
P for trend		<0.01		0.01		0.25		0.06		0.08		0.33

Lung cancer												
1st Quartile	3	1.0 (Ref)	3	1.0 (Ref)	2	1.0 (Ref)	46	1.0 (Ref)	43	1.0 (Ref)	39	1.0 (Ref)
2nd Quartile	13	0.62 (0.17, 2.17)	12	0.57 (0.16, 2.06)	12	0.90 (0.20, 4.09)	42	0.99 (0.65, 1.51)	40	0.97 (0.63, 1.50)	40	1.11 (0.66, 1.87)
3rd Quartile	18	0.41 (0.12, 1.39)	17	0.39 (0.11, 1.36)	16	0.77 (0.16, 3.68)	28	0.81 (0.50, 1.30)	27	0.73 (0.44, 1.19)	27	0.92 (0.46, 1.83)
4th Quartile	39	0.73 (0.22, 2.39)	38	0.55 (0.16, 1.85)	38	1.66 (0.32, 8.72)	20	0.70 (0.41, 1.19)	19	0.57† (0.32, 1.01)	19	0.76 (0.32, 1.82)
P for trend		0.54		0.80		0.17		0.14		0.04		0.57
Leukemia												
1st Quartile	0	--	0	--	0	--	13	1.0 (Ref)	13	1.0 (Ref)	13	1.0 (Ref)
2nd Quartile	12	1.0 (Ref)	12	1.0 (Ref)	12	1.0 (Ref)	13	1.26 (0.58, 2.73)	12	1.12 (0.51, 2.47)	12	1.56 (0.63, 3.85)
3rd Quartile	16	0.71 (0.33, 1.50)	16	0.72 (0.34, 1.53)	16	0.81 (0.34, 1.93)	7	0.91 (0.36, 2.30)	7	0.83 (0.32, 2.12)	7	1.70 (0.51, 5.69)
4th Quartile	26	1.03 (0.52, 2.06)	24	1.00 (0.49, 2.05)	24	1.09 (0.39, 3.08)	11	1.71 (0.76, 3.86)	10	1.33 (0.56, 3.20)	10	3.83 (0.92, 15.89)
P for trend		0.34		0.40		0.40		0.33		0.70		0.09
Non-Hodgkin lymphoma												
1st Quartile	0	--	0	--	0	--	16	1.0 (Ref)	15	1.0 (Ref)	15	1.0 (Ref)
2nd Quartile	8	1.0 (Ref)	8	1.0 (Ref)	8	1.0 (Ref)	10	0.76 (0.34, 1.68)	9	0.70 (0.30, 1.60)	9	0.53 (0.20, 1.40)
3rd Quartile	13	0.88 (0.36, 2.12)	13	0.88 (0.36, 2.13)	13	0.89 (0.32, 2.51)	9	0.90 (0.39, 2.05)	9	0.90 (0.39, 2.10)	9	0.65 (0.21, 2.01)
4th Quartile	32	1.96† (0.90, 4.28)	32	1.89 (0.85, 4.19)	32	1.37 (0.43, 4.39)	11	1.32 (0.60, 2.86)	11	1.31 (0.58, 2.98)	11	0.95 (0.25, 3.59)
P for trend		<0.01		0.01		0.25		0.53		0.53		0.95
Hematological cancers												
1st Quartile	0	--	0	--	0	--	30	1.0 (Ref)	29	1.0 (Ref)	29	1.0 (Ref)
2nd Quartile	21	1.0 (Ref)	21	1.0 (Ref)	21	1.0 (Ref)	24	0.99 (0.58, 1.71)	22	0.92 (0.52, 1.60)	22	0.95 (0.50, 1.84)
3rd Quartile	30	0.77 (0.44, 1.34)	30	0.77 (0.44, 1.35)	30	0.84 (0.44, 1.61)	16	0.88 (0.48, 1.63)	16	0.87 (0.46, 1.61)	16	1.03 (0.45, 2.35)
4th Quartile	58	1.34 (0.81, 2.22)	56	1.30 (0.77, 2.18)	56	1.18 (0.55, 2.52)	23	1.52 (0.88, 2.64)	22	1.40 (0.78, 2.52)	22	1.93 (0.74, 5.08)
P for trend		0.02		0.03		0.18		0.24		0.38		0.23

Note: Hazard ratios (HRs) and 95% confidence intervals (CIs) from Cox regression models are presented

[1]waist circumference quartile 1: 67.9 cm – 81.9 cm (males), 55.9 cm – 81.9 cm (females)

[1]waist circumference quartile 2: 82.0 cm – 91.8 cm (males), 82.0 cm – 91.8 cm (females)

[1]waist circumference quartile 3: 91.9 cm – 102.9 cm (males), 91.9 cm – 102.9 cm (females)

[1]waist circumference quartile 4: 103.0 cm – 149.9 cm (males), 103.0 cm – 149.9 cm (females)

[2]Adjusted for: age (continuous), sex (for non sex-specific cancers), ethnicity (white/other), marital status (married or living with someone/divorced, separated, or widowed/single, never married), highest level of education (high school or less/some post-high school education/post-high school certificate or degree), total household income ($0 to $49,999/$50,000 to $99,999/≥ $100,000), geographical area of residence (urban/rural), smoking status (current/former/never), alcohol consumption (grams of ethanol per day), energy intake (kilocalories per day), total physical activity (MET-hours per week), history of diabetes (yes/no), family history of cancer (yes/no), pack-years of cigarettes (lung cancer only), fibre intake (grams per day) (colon cancer only), menopausal status (pre-menopause/post-menopause) (breast cancer), years of birth control use (0 to 5 years/>5 years), (breast and endometrial cancers), history of breast cancer screening (yes/no) (breast cancer only), history of colon cancer screening (yes/no) (colon cancer only), and history of prostate cancer screening (yes/no) (prostate cancer only).

[3]Adjusted for: all variables in multivariate-analysis[2] as well as BMI (≥18.5 kg/m^2 and < 25 kg/m^2/ = ≥ 25 kg/m^2 and < 30 kg/m^2/ = ≥ 30 kg/m^2).

**p<0.01, *p<0.05, †p<0.1

Supplementary Table 6. Results from analyses of waist-to-hip ratio in quartiles [1] and cancer incidence for cancers occurring greater than two years after baseline data collection (n = 26060)

	MALES (n=9 764)				FEMALES (n=16 296)			
	Cases	Latency Multivariate-adjusted[2]	Cases	Latency Multivariate-adjusted[3]	Cases	Latency Multivariate-adjusted[2]	Cases	Latency Multivariate-adjusted[3]
All-cancer								
1st Quartile	143	1.0 (Ref)	141	1.0 (Ref)	220	1.0 (Ref)	217	1.0 (Ref)
2nd Quartile	187	1.09 (0.87, 1.35)	186	1.07 (0.85, 1.34)	278	1.17† (0.98, 1.40)	275	1.15 (0.96, 1.37)
3rd Quartile	237	1.20† (0.98, 1.49)	237	1.15 (0.91, 1.44)	300	1.21* (1.02, 1.45)	297	1.15 (0.96, 1.38)
4th Quartile	290	1.39** (1.13, 1.71)	290	1.27* (1.00, 1.60)	343	1.24* (1.03, 1.47)	343	1.13 (0.94, 1.37)
P for trend		<0.01		0.03		0.02		0.27
Prostate cancer								
1st Quartile	60	1.0 (Ref)	59	1.0 (Ref)	--	--	--	--
2nd Quartile	69	0.96 (0.68, 1.36)	69	0.97 (0.68, 1.38)	--	--	--	--
3rd Quartile	89	1.09 (0.78, 1.52)	89	1.10 (0.77, 1.57)	--	--	--	--
4th Quartile	120	1.46* (1.06, 2.02)	120	1.49 (1.04, 2.14)	--	--	--	--
P for trend		0.01		0.01		--		--
Breast cancer – Premenopausal								
1st Quartile	--	--	--	--	45	1.0 (Ref)	44	1.0 (Ref)
2nd Quartile	--	--	--	--	49	1.23 (0.82, 1.85)	48	1.28 (0.84, 1.93)
3rd Quartile	--	--	--	--	48	1.35 (0.89, 2.03)	47	1.47† (0.96, 2.27)
4th Quartile	--	--	--	--	28	0.93 (0.57, 1.50)	28	1.09 (0.64, 1.85)
P for trend		--		--		0.91		0.42
Breast cancer – Postmenopausal								
1st Quartile	--	--	--	--	42	1.0 (Ref)	42	1.0 (Ref)
2nd Quartile	--	--	--	--	68	1.38 (0.94, 2.02)	67	1.33 (0.90, 1.97)
3rd Quartile	--	--	--	--	55	1.00 (0.67, 1.50)	55	0.95 (0.63, 1.44)
4th Quartile	--	--	--	--	76	1.13 (0.79, 1.72)	76	1.05 (0.69, 1.59)
P for trend		--		--		0.94		0.67
Endometrial cancer								
1st Quartile	--	--	--	--	14	1.0 (Ref)	14	1.0 (Ref)
2nd Quartile	--	--	--	--	17	1.21 (0.60, 2.47)	17	0.97 (0.48, 1.99)
3rd Quartile	--	--	--	--	31	2.24* (1.18, 4.24)	30	1.37 (0.70, 2.67)
4th Quartile	--	--	--	--	34	2.22* (1.16, 4.23)	34	1.10 (0.55, 2.20)
P for trend		--		--		<0.01		0.65
Colon cancer								
1st Quartile	9	1.0 (Ref)	9	1.0 (Ref)	17	1.0 (Ref)	17	1.0 (Ref)
2nd Quartile	12	1.05 (0.44, 2.50)	12	0.89 (0.37, 2.15)	24	1.29 (0.69, 2.41)	24	1.14 (0.61, 2.13)
3rd Quartile	22	1.60 (0.73, 3.50)	22	1.16 (0.51, 2.67)	23	1.18 (0.63, 2.23)	22	0.92 (0.47, 1.77)
4th Quartile	29	1.90 (0.88, 4.11)	29	1.22 (0.53, 2.84)	26	1.17 (0.62, 2.21)	26	0.90 (0.46, 1.76)
P for trend		0.04		0.45		0.75		0.58
Lung cancer								
1st Quartile	8	1.0 (Ref)	7	1.0 (Ref)	17	1.0 (Ref)	15	1.0 (Ref)
2nd Quartile	15	1.56 (0.66, 3.70)	14	1.88 (0.74, 4.77)	28	1.18 (0.64, 2.17)	28	1.38 (0.73, 2.61)
3rd Quartile	15	1.14 (0.48, 2.70)	15	1.58 (0.60, 4.15)	31	1.03 (0.56, 1.87)	31	1.24 (0.66, 2.35)
4th Quartile	24	1.15 (0.50, 2.65)	24	1.68 (0.63, 4.44)	40	0.90 (0.49, 1.62)	40	1.15 (0.60, 2.22)
P for trend		0.92		0.49		0.48		0.94
Leukemia								
1st Quartile	10	1.0 (Ref)	10	1.0 (Ref)	4	1.0 (Ref)	4	1.0 (Ref)
2nd Quartile	10	0.89 (0.37, 2.16)	10	0.91 (0.37, 2.23)	7	1.70 (0.50, 5.84)	7	1.81 (0.53, 6.25)
3rd Quartile	15	1.26 (0.56, 2.84)	15	1.28 (0.53, 3.07)	11	2.60 (0.82, 8.23)	11	3.05† (0.94, 9.89)
4th Quartile	10	0.84 (0.34, 2.08)	10	0.83 (0.30, 2.28)	13	2.84† (0.90, 8.92)	13	3.76* (1.12, 12.61)
P for trend		0.93		0.91		0.05		0.02
Non-Hodgkin lymphoma								
1st Quartile	3	1.0 (Ref)	3	1.0 (Ref)	8	1.0 (Ref)	8	1.0 (Ref)
2nd Quartile	17	5.00* (1.46, 17.14)	17	4.52* (1.29, 15.78)	9	1.02 (0.39, 2.67)	9	0.92 (0.35, 2.43)
3rd Quartile	14	3.71* (1.05, 13.03)	14	2.84 (0.77, 10.53)	9	0.96 (0.37, 2.50)	9	0.79 (0.29, 2.12)
4th Quartile	14	3.42† (0.96, 12.16)	14	2.17 (0.57, 8.35)	14	1.29 (0.52, 3.17)	14	0.97 (0.37, 2.55)
P for trend		0.27		0.92		0.59		0.93

Hematological cancers								
1st Quartile	14	1.0 (Ref)	14	1.0 (Ref)	13	1.0 (Ref)	13	1.0 (Ref)
2nd Quartile	27	1.72 (0.90, 3.30)	27	1.67 (0.86, 3.24)	17	1.26 (0.61, 2.60)	17	1.23 (0.59, 2.55)
3rd Quartile	30	1.76† (0.93, 3.36)	30	1.59 (0.80, 3.17)	20	1.41 (0.70, 2.85)	20	1.36 (0.66, 2.82)
4th Quartile	24	1.37 (0.70, 2.70)	24	1.11 (0.52, 2.34)	28	1.80† (0.91, 3.54)	28	1.73 (0.84, 3.59)
P for trend		0.49		0.92		0.08		0.13

Note: Hazard ratios (HRs) and 95% confidence intervals (CIs) from Cox regression models are presented

[1] Quartiles of WHR for males: 1st Quartile = 0.74 - 0.92, 2nd Quartile = 0.93 - 0.97, 3rd Quartile = 0.97 – 1.02, 4th quartile = 1.02 – 1.50

Quartiles of WHR for females: 1st Quartile = 0.57 - 0.78, 2nd Quartile = 0.78 – 0.82, 3rd Quartile = 0.82 – 0.87, 4th Quartile = 0.87 – 1.46

[2]Adjusted for: age (continuous), ethnicity (white/other), marital status (married or living with someone/divorced, separated, or widowed/single, never married), highest level of education (high school or less/some post-high school education/post-high school certificate or degree), total household income ($0 to $49,999/$50,000 to $99,999/≥ $100,000), geographical area of residence (urban/rural), smoking status (current/former/never), alcohol consumption (grams of ethanol per day), energy intake (kilocalories per day), total physical activity (MET-hours per week), history of diabetes (yes/no), family history of cancer (yes/no), pack-years of cigarettes (lung cancer only), fibre intake (grams per day) (colon cancer only), menopausal status (pre-menopause/post-menopause) (breast and endometrial cancers), years of birth control use (0 to 5 years/>5 years), (breast and endometrial cancers), history of breast cancer screening (yes/no) (breast cancer only), history of colon cancer screening (yes/no) (colon cancer only), and history of prostate cancer screening (yes/no) (prostate cancer only)

[3]Adjusted for: all variables in multivariate-analysis[2] as well as BMI (≥18.5 kg/m^2 and < 25 kg/m^2/ = ≥ 25 kg/m^2 and < 30 kg/m^2/ = ≥ 30 kg/m^2).

**p<0.01, *p<0.05, †p<0.1

Supplementary Table 7. Results from analyses of waist-to-height-ratio in quartiles[1] and cancer incidence (n=26 478)

	MALES (n=9 981)						FEMALES (n=16 497)					
	Cases	Age-adjusted	Cases	Multivariate-adjusted[2]	Cases	Multivariate-adjusted[3]	Cases	Age-adjusted	Cases	Multivariate-adjusted[2]	Cases	Multivariate-adjusted[3]
All-cancer												
1st Quartile	81	1.0 (Ref)	79	1.0 (Ref)	77	1.0 (Ref)	345	1.0 (Ref)	333	1.0 (Ref)	323	1.0 (Ref)
2nd Quartile	206	0.90 (0.70, 1.17)	202	0.91 (0.71, 1.18)	202	0.91 (0.69, 1.21)	317	1.10 (0.94, 1.28)	301	1.08 (0.93, 1.27)	300	1.14 (0.95, 1.37)
3rd Quartile	349	1.04 (0.81, 1.33)	341	1.05 (0.82, 1.34)	341	1.02 (0.75, 1.39)	274	1.07 (0.91, 1.26)	262	1.06 (0.90, 1.25)	262	1.13 (0.91, 1.42)
4th Quartile	372	1.23 (0.96, 1.57)	364	1.23 (0.95, 1.57)	364	1.12 (0.79, 1.57)	410	1.34** (1.16, 1.55)	395	1.31** (1.25, 1.53)	394	1.31† (1.01, 1.70)
P for trend		<0.01		<0.01		0.23		<0.01		<0.01		0.06
Prostate cancer												
1st Quartile	30	1.0 (Ref)	29	1.0 (Ref)	28	1.0 (Ref)	--	--	--	--	--	--
2nd Quartile	81	0.94 (0.61, 1.42)	81	0.95 (0.62, 1.45)	81	0.96 (0.87, 1.52)	--	--	--	--	--	--
3rd Quartile	157	1.22 (0.82, 1.81)	153	1.20 (0.80, 1.79)	153	1.22 (0.74, 2.00)	--	--	--	--	--	--
4th Quartile	132	1.12 (0.75, 1.68)	129	1.16 (0.77, 1.75)	129	1.22 (0.70, 2.12)	--	--	--	--	--	--
P for trend		0.23		0.18		0.27	--	--	--	--	--	--
Breast cancer – Premenopausal												
1st Quartile	--	--	--	--	--	--	85	1.0 (Ref)	82	1.0 (Ref)	79	1.0 (Ref)
2nd Quartile	--	--	--	--	--	--	45	0.91 (0.63, 1.31)	44	0.90 (0.63, 1.31)	44	1.16 (0.76, 1.77)
3rd Quartile	--	--	--	--	--	--	31	0.88 (0.58, 1.34)	28	0.82 (0.53, 1.26)	28	1.33 (0.74, 2.39)
4th Quartile	--	--	--	--	--	--	33	0.92 (0.61, 1.38)	31	0.85 (0.55, 1.31)	31	1.68 (0.79, 3.57)
P for trend	--	--	--	--	--	--		0.59		0.35		0.18
Breast cancer – Postmenopausal												
1st Quartile	--	--	--	--	--	--	56	1.0 (Ref)	52	1.0 (Ref)	51	1.0 (Ref)
2nd Quartile	--	--	--	--	--	--	73	1.25 (0.88, 1.77)	72	1.35 (0.94, 1.93)	72	1.45† (0.97, 2.16)
3rd Quartile	--	--	--	--	--	--	66	1.72 (0.82, 1.68)	62	1.24 (0.85, 1.79)	62	1.28 (0.79, 2.10)
4th Quartile	--	--	--	--	--	--	91	1.29 (0.92, 1.80)	88	1.40 (0.97, 2.00)	88	1.20 (0.68, 2.12)
P for trend	--	--	--	--	--	--		0.21		0.12		0.68
Endometrial cancer												
1st Quartile	--	--	--	--	--	--	19	1.0 (Ref)	19	1.0 (Ref)	19	1.0 (Ref)
2nd Quartile	--	--	--	--	--	--	18	1.22 (0.64, 2.34)	14	1.01 (0.50, 2.02)	14	0.70 (0.30, 1.59)
3rd Quartile	--	--	--	--	--	--	19	1.52 (0.80, 2.89)	18	1.54 (0.80, 2.97)	18	0.71 (0.28, 1.84)
4th Quartile	--	--	--	--	--	--	66	4.52** (2.68, 7.60)	60	4.19** (2.42, 7.23)	59	1.29 (0.47, 3.49)
P for trend	--	--	--	--	--	--		<0.01		<0.01		0.28
Colon cancer												
1st Quartile	4	1.0 (Ref)	4	1.0 (Ref)	4	1.0 (Ref)	21	1.0 (Ref)	21	1.0 (Ref)	21	1.0 (Ref)
2nd Quartile	11	0.98 (0.31, 3.08)	11	0.96 (0.30, 3.03)	11	0.82 (0.24, 2.75)	27	1.49 (084, 2.64)	26	1.43 (0.80, 2.55)	25	1.02 (0.52, 2.00)
3rd Quartile	32	1.94 (0.68, 5.53)	32	1.87 (0.65, 5.35)	32	1.28 (0.36, 4.56)	27	1.64 (0.93, 2.94)	27	1.64 (0.92, 2.94)	27	1.13 (0.52, 2.42)
4th Quartile	35	2.36 (0.83, 6.71)	34	2.01 (0.70, 5.82)	34	0.99 (0.25, 3.93)	33	1.67 (0.95, 2.91)	33	1.60 (0.90, 2.84)	33	1.26 (0.52, 3.07)

P for trend		<0.01		0.03		0.89		0.08		0.11		0.57
Lung cancer												
1st Quartile	4	1.0 (Ref)	4	1.0 (Ref)	3	1.0 (Ref)	36	1.0 (Ref)	34	1.0 (Ref)	30	1.0 (Ref)
2nd Quartile	18	1.45 (0.49, 4.28)	16	1.34 (0.45, 4.05)	16	2.07 (0.59, 7.23)	31	0.87 (0.54, 1.41)	29	0.91 (0.55, 1.49)	29	1.09 (0.63, 1.90)
3rd Quartile	19	0.98 (0.33, 2.90)	19	0.98 (0.33, 2.93)	19	2.06 (0.53, 8.00)	36	1.03 (0.65, 1.65)	35	0.96 (0.59, 1.56)	35	1.31 (0.69, 2.49)
4th Quartile	32	1.75 (0.61, 4.99)	31	1.25 (0.43, 3.65)	31	3.58† (0.84, 15.31)	33	0.76 (0.47, 1.22)	31	0.62 (0.37, 1.03)	31	0.97 (0.43, 2.22)
P for trend		0.27		0.88		0.09		0.38		0.09		0.84
Leukemia												
1st Quartile	4	1.0 (Ref)	4	1.0 (Ref)	4	1.0 (Ref)	10	1.0 (Ref)	10	1.0 (Ref)	10	1.0 (Ref)
2nd Quartile	14	1.34 (0.38, 4.07)	14	1.43 (0.47, 4.38)	14	1.59 (0.49, 5.12)	14	1.65 (0.73, 3.74)	13	1.46 (0.64, 3.35)	13	1.84 (0.74, 4.58)
3rd Quartile	13	0.89 (0.29, 2.75)	13	0.98 (0.47, 4.40)	13	1.23 (0.32, 4.75)	6	0.79 (2.85, 2.20)	6	0.73 (0.26, 2.03)	6	1.21 (0.34, 4.23)
4th Quartile	23	1.81 (0.61, 5.33)	21	1.89 (0.47, 4.39)	21	2.66 (0.61, 11.63)	14	1.53 (0.67, 3.51)	13	1.19 (0.50, 2.87)	13	2.71 (0.66, 11.07)
P for trend		0.24		0.28		0.23		0.59		0.98		0.26
Non-Hodgkin lymphoma												
1st Quartile	3	1.0 (Ref)	3	1.0 (Ref)	3	1.0 (Ref)	12	1.0 (Ref)	11	1.0 (Ref)	11	1.0 (Ref)
2nd Quartile	13	1.70 (0.48, 5.99)	13	1.80 (0.51, 6.37)	13	1.55 (0.41, 5.88)	11	1.05 (0.46, 2.40)	10	1.00 (0.42, 2.38)	10	0.81 (0.30, 2.22)
3rd Quartile	12	1.14 (0.32, 4.08)	12	1.19 (0.33, 4.30)	12	0.78 (0.17, 3.52)	8	0.85 (0.34, 2.09)	8	0.88 (0.35, 2.23)	8	0.64 (0.19, 2.15)
4th Quartile	25	2.76 (0.82, 9.31)	25	2.71 (0.79, 9.25)	25	1.05 (0.21, 5.30)	16	1.40 (0.65, 3.01)	16	1.43 (0.64, 3.20)	16	0.97 (0.24, 3.80)
P for trend		0.05		0.08		0.76		0.46		0.42		0.92
Hematological cancers												
1st Quartile	8	1.0 (Ref)	8	1.0 (Ref)	8	1.0 (Ref)	23	1.0 (Ref)	22	1.0 (Ref)	22	1.0 (Ref)
2nd Quartile	28	1.36 (0.62, 3.00)	28	1.45 (0.66, 3.19)	28	1.42 (0.62, 3.27)	25	1.27 (0.72, 2.25)	23	1.18(0.66, 2.14)	23	1.23 (0.63, 2.39)
3rd Quartile	25	0.88 (0.39, 1.97)	25	0.94 (0.42, 2.11)	25	0.86 (0.33, 2.27)	15	0.85 (0.44, 1.65)	15	0.86 (0.44, 1.66)	15	0.96 (0.41, 2.24
4th Quartile	48	1.96 (0.92, 4.20)	46	1.98 (0.91, 4.29)	46	1.47 (0.51, 4.22)	31	1.46 (0.84, 2.54)	30	1.36 (0.76, 2.43)	30	1.70 (0.65, 4.47)
P for trend		0.05		0.08		0.66		0.32		0.45		0.39

Note: Hazard ratios (HRs) and 95% confidence intervals (CIs) from Cox regression models are presented

[1] waist-to-height-ratio quartile 1: 0.37 – 0.52 (males), 0.34 – 0.47 (females)

[1] waist-to-height-ratio quartile 2: 0.52 – 0.60 (males), 0.47 – 0.53 (females)

[1] waist-to-height-ratio quartile 3: 0.60 – 0.77 (males), 0.53 – 0.82 (females)

[1] waist-to-height-ratio quartile 4: 0.77 – 1.03 (males), 0.82 – 1.12 (females)

[2]Adjusted for: age (continuous), sex (for non sex-specific cancers), ethnicity (white/other), marital status (married or living with someone/divorced, separated, or widowed/single, never married), highest level of education (high school or less/some post-high school education/post-high school certificate or degree), total household income ($0 to $49,999/$50,000 to $99,999/≥ $100,000), geographical area of residence (urban/rural), smoking status (current/former/never), alcohol consumption (grams of ethanol per day), energy intake (kilocalories per day), total physical activity (MET-hours per week), history of diabetes (yes/no), family history of cancer (yes/no), pack-years of cigarettes (lung cancer only), fibre intake (grams per day) (colon cancer only), menopausal status (pre-menopause/post-menopause) (breast cancer), years of birth control use (0 to 5 years/>5 years), (breast and endometrial cancers), history of breast cancer screening (yes/no) (breast cancer only), history of colon cancer screening (yes/no) (colon cancer only), and history of prostate cancer screening (yes/no) (prostate cancer only).

[3]Adjusted for: all variables in multivariate-analysis[2] as well as BMI ($\geq$18.5 kg/m^2 and < 25 kg/m^2/ = ≥ 25 kg/m^2 and < 30 kg/m^2/ = ≥ 30 kg/m^2).

**p<0.01, *p<0.05, †p<0.1

Supplementary Table 8. Results from analyses of BMI categories[1] and cancer incidence among ATP participants with BMI ≥18.5 kg/m^2 by smoking status (n=25 588)

	All Participants	Smoking Status				
	Cases	Never Smoker	Cases	Former Smoker	Cases	Current Smoker
All-cancer						
Normal	242	1.00 (Ref)	206	1.18† (0.98, 1.42)	184	2.04** (1.68, 2.47)
Overweight	341	1.19* (1.01, 1.40)	369	1.25** (1.06, 1.47)	179	1.65** (1.36, 2.01)
Obese	263	1.41** (1.19, 1.69)	365	1.59** (1.35, 1.87)	123	1.97** (1.58, 2.45)
P for trend		<0.01		<0.01		<0.01
Lung cancer						
Normal	6	1.00 (Ref)	16	3.39** (1.33, 8.68)	47	21.76** (9.24, 51.25)
Overweight	7	0.88 (0.30, 2.63)	26	2.82* (1.16, 6.87)	45	15.81** (6.71, 37.26)
Obese	5	0.90 (0.27, 2.96)	24	3.17* (1.29, 7.80)	18	10.58** (4.15, 26.92)
P for trend		0.16		0.16		0.16

Note: Hazard ratios (HRs) and 95% confidence intervals (CIs) from Cox regression models are presented
[1]Normal = ≥18.5 kg/m^2 and < 25 kg/m^2; Overweight = ≥ 25 kg/m^2; Obese = ≥ 30 kg/m^2. Those with a BMI <18.5 kg/m^2 were excluded from these analyses.
[2]Adjusted for: age (continuous), ethnicity (white/other), marital status (married or living with someone/divorced, separated, or widowed/single, never married), highest level of education (high school or less/some post-high school education/post-high school certificate or degree), total household income ($0 to $49,999/$50,000 to $99,999/≥ $100,000), geographical area of residence (urban/rural), alcohol consumption (grams of ethanol per day), energy intake (kilocalories per day), total physical activity (MET-hours per week), history of diabetes (yes/no), family history of cancer (yes/no)
**p<0.01, *p<0.05, †p<0.1

Supplementary Table 9. Results from analyses of waist circumference[1] and cancer incidence among by smoking status (n=25 715)

	All Participants Smoking Status					
	Cases	Never Smoker	Cases	Former Smoker	Cases	Current Smoker
All-cancer						
Below	443	1.00 (Ref)	391	1.09 (0.95, 1.25)	274	1.73** (1.49, 2.02)
Above	400	1.21** (1.06, 1.39)	549	1.39** (1.22, 1.58)	221	1.71** (1.45, 2.02)
P for trend		<0.01		<0.01		<0.01
Lung cancer						
Below	7	1.00 (Ref)	20	3.17** (1.34, 7.49)	70	29.29** (13.39, 64.08)
Above	11	1.74 (0.67, 4.49)	46	5.49** (2.47, 12.21)	46	19.95** (8.94, 44.55)
P for trend		<0.01		<0.01		<0.01

Note: Hazard ratios (HRs) and 95% confidence intervals (CIs) from Cox regression models are presented
[1]waist circumference below guidelines: <102 cm for men, < 88 cm for women
[1]waist circumference above guidelines: ≥102 cm for men, ≥88 cm for women
[2]Adjusted for: age (continuous), ethnicity (white/other), marital status (married or living with someone/divorced, separated, or widowed/single, never married), highest level of education (high school or less/some post-high school education/post-high school certificate or degree), total household income ($0 to $49,999/$50,000 to $99,999/≥ $100,000), geographical area of residence (urban/rural), alcohol consumption (grams of ethanol per day), energy intake (kilocalories per day), total physical activity (MET-hours per week), history of diabetes (yes/no), family history of cancer (yes/no)
**p<0.01, *p<0.05, †p<0.1

Supplementary Table 10. Results from analyses of waist-to-hip ratio in quartiles[1] and cancer incidence among by smoking status (n=25 823)

	All Participants Smoking Status					
	Cases	Never Smoker	Cases	Former Smoker	Cases	Current Smoker
All-cancer						
1st Quartile	212	1.00 (Ref)	141	0.94 (0.76, 1.17)	105	1.83** (1.45, 2.32)
2nd Quartile	199	0.98 (0.81, 1.19)	208	1.22* (1.00, 1.47)	112	1.66** (1.32, 2.09)
3rd Quartile	208	1.06 (0.87, 1.29)	248	1.33** (1.10, 1.60)	123	1.62** (1.29, 2.03)
4th Quartile	229	1.47** (1.22, 1.78)	347	1.45** (1.21, 1.72)	159	1.87** (1.51, 2.31)
P for trend		<0.01		<0.01		<0.01
Lung cancer						
1st Quartile	3	1.00 (Ref)	4	1.78 (0.40, 7.94)	28	39.16** (11.85, 129.37)
2nd Quartile	2	0.60 (0.10, 3.61)	19	6.58** (1.95, 22.26)	31	33.33** (10.15, 109.43)
3rd Quartile	7	2.18 (0.56, 8.45)	17	5.04* (1.47, 17.25)	26	22.39** (6.75, 74.30)
4th Quartile	6	2.37 (0.59, 9.51)	26	5.38** (1.62, 17.87)	31	21.50** (6.52, 70.88)
P for trend		<0.01		<0.01		<0.01

Note: Hazard ratios (HRs) and 95% confidence intervals (CIs) from Cox regression models are presented

[1]Quartiles of WHR for males: 1st quartile = 0.7394 - 0.9257, 2nd quartile = 0.9258 - 0.9736, 3rd quartile = 0.9737 – 1.0225, 4th quartile = 1.0226 – 1.4983.

[1]Quartiles of WHR for females: 1st quartile = 0.5748 - 0.7818, 2nd quartile = 0.7819 – 0.8289, 3rd quartile = 0.8290 – 0.8794, 4th quartile = 0.8795 – 1.4587.

[2]Adjusted for: age (continuous), ethnicity (white/other), marital status (married or living with someone/divorced, separated, or widowed/single, never married), highest level of education (high school or less/some post-high school education/post-high school certificate or degree), total household income ($0 to $49,999/$50,000 to $99,999/≥ $100,000), geographical area of residence (urban/rural), alcohol consumption (grams of ethanol per day), energy intake (kilocalories per day), total physical activity (MET-hours per week), history of diabetes (yes/no), family history of cancer (yes/no)

**p<0.01, *p<0.05, †p<0.1
